# The ubiquitin ligase HUWE1 enhances WNT signaling by antagonizing destruction complex-mediated β-catenin degradation and through a mechanism independent of changes in β-catenin abundance

**DOI:** 10.1371/journal.pgen.1011677

**Published:** 2025-05-27

**Authors:** Joseph K. McKenna, Yalan Wu, Praveen Sonkusre, Caleb K. Sinclear, Raj Chari, Andres M. Lebensohn

**Affiliations:** 1 Laboratory of Cellular and Molecular Biology, Center for Cancer Research, National Cancer Institute, National Institutes of Health, Bethesda, Maryland, United States of America; 2 Genome Modification Core, Laboratory Animal Sciences Program, Frederick National Lab for Cancer Research, Frederick, Maryland, United States of America; Shanghai Institute of Biochemistry and Cell Biology, Chinese Academy of Sciences, CHINA

## Abstract

WNT/β-catenin signaling is mediated by the transcriptional coactivator β-catenin (CTNNB1). CTNNB1 abundance is regulated by phosphorylation and proteasomal degradation, promoted by a destruction complex composed of the scaffold proteins APC and AXIN1 or AXIN2, and the kinases casein kinase 1α (CSNK1A1) and GSK3A or GSK3B. Loss of CSNK1A1 increases CTNNB1 abundance, resulting in hyperactive WNT signaling. Previously, we demonstrated that the HECT domain E3 ubiquitin ligase HUWE1 is necessary for hyperactive WNT signaling in HAP1 haploid human cells lacking CSNK1A1. Here, we investigated the mechanism underlying this requirement. In HAP1 cells lacking CSNK1A1, GSK3A/GSK3B still phosphorylated a fraction of CTNNB1, promoting its degradation. HUWE1 loss enhanced GSK3A/GSK3B-dependent CTNNB1 phosphorylation, further reducing CTNNB1 abundance. However, the reduction in CTNNB1 caused by HUWE1 loss was smaller than the reduction in WNT target gene transcription. To test whether the reduction in WNT signaling caused by HUWE1 loss resulted from reduced CTNNB1 alone, we engineered the endogenous *CTNNB1* locus in HAP1 cells to encode a CTNNB1 variant insensitive to destruction complex-mediated phosphorylation and degradation. HUWE1 loss in these cells did not change CTNNB1 abundance but still reduced WNT signaling, demonstrating that another mechanism was at play. Genetic interaction and overexpression analyses revealed that the reduction in WNT signaling caused by HUWE1 loss required not only GSK3A or GSK3B, but also APC and AXIN1. Therefore, in HAP1 cells lacking CSNK1A1, a residual destruction complex containing APC, AXIN1 and GSK3A or GSK3B downregulates WNT signaling by phosphorylating and targeting CTNNB1 for degradation, and HUWE1 enhances WNT signaling by antagonizing this activity. Regulation of WNT signaling by HUWE1 also requires its ubiquitin ligase activity. We conclude that HUWE1 enhances WNT/CTNNB1 signaling through two mechanisms, one that antagonizes destruction complex-mediated CTNNB1 degradation and another that is independent of changes in CTNNB1 abundance. Coordinated regulation of CTNNB1 abundance and a second signaling step by HUWE1 would be an efficient way to control WNT signaling output, enabling sensitive and robust activation of the pathway.

## Author summary

The WNT pathway is a conserved cell signaling system with diverse functions in embryonic development and adult tissue homeostasis. Dysregulation of WNT signaling drives many types of cancer. Over four decades of research have revealed a great deal about how the core components of the WNT pathway regulate signaling, but much less is known about additional regulatory layers superimposed on the core signaling module. In this study we present an example of such regulation by the ubiquitin ligase HUWE1, a protein that attaches ubiquitin to other proteins. Phosphorylation of the transcriptional coactivator β-catenin by a protein complex called the ‘destruction complex’ tags β-catenin for degradation. Control of β-catenin abundance by the destruction complex is considered the main regulated step in WNT signaling. We demonstrate that HUWE1 enhances WNT signaling through two distinct mechanisms. First, HUWE1 antagonizes the phosphorylation and degradation of β-catenin by the destruction complex, thereby increasing β-catenin abundance. Second, HUWE1 enhances WNT signaling through a mechanism independent from changes in β-catenin abundance. Our work therefore reveals a new role for HUWE1 controlling the main regulated step in WNT signaling – β-catenin phosphorylation and degradation by the destruction complex – and most likely a downstream mechanism. This coordinated regulation may enable sensitive and robust activation of the WNT pathway.

## Introduction

During embryonic development and tissue homeostasis, WNT/β-catenin signaling orchestrates cellular processes that control tissue patterning and morphogenesis, cell fate specification, and stem cell self-renewal among many other functions [1,2]. Mutations in WNT signaling pathway components can drive tumorigenesis of many cancer types, most notably colorectal cancer [3,4]. At the heart of the WNT/β-catenin signaling pathway, the destruction complex controls the abundance of the transcriptional coactivator β-catenin (CTNNB1) by regulating its degradation through the ubiquitin/proteasome system. The destruction complex is comprised of a set of core components, including the scaffold proteins adenomatous polyposis coli (APC) and axis inhibition protein 1 (AXIN1) or 2 (AXIN2), and the kinases casein kinase 1α (CSNK1A1) and glycogen synthase kinase 3α (GSK3A) or β (GSK3B) [5]. In the absence of signals initiated by secreted WNT ligands, CSNK1A1 phosphorylates CTNNB1 at serine (S) 45 [6,7], priming it for further sequential phosphorylation of threonine (T) 41, S37 and S33 by GSK3A and/or GSK3B [6,8] (we refer to residues S33, S37, T41 and S45 as the CTNNB1 phosphodegron). When phosphorylated, residues S33 and S37 create a recognition site for the Skp, Cullin, F-box containing ubiquitin ligase complex SCF^βTrCP^ [9,10], which ubiquitylates CTNNB1 and targets it for proteasomal degradation [5]. Therefore, CTNNB1 abundance is kept low and WNT-dependent transcriptional programs are repressed. Binding of WNT ligands to the cell surface receptors frizzled (FZD) and LDL receptor related proteins 5 (LRP5) or 6 (LRP6) triggers the recruitment of dishevelled (DVL) and at least some destruction complex components to FZD and LRP5/6. Formation of this receptor complex, or signalosome, downregulates the destruction complex [11,12] and results in accumulation of non-phosphorylated CTNNB1. CTNNB1 enters the nucleus, where it forms a complex with transcription factors of the T cell factor (TCF)/lymphoid enhancer factor (LEF) family and other coactivators to drive WNT target gene transcription [13].

This description of WNT/CTNNB1 signaling omits many additional regulatory mechanisms superimposed on the core pathway that control the abundance, interactions, and subcellular localization of many components of the pathway. Such additional regulatory mechanisms tune WNT responses in diverse biological contexts, expand the functional repertoire of the pathway, and may represent potential sites of therapeutic intervention in WNT-driven cancers. Classical genetic approaches have been very successful at discovering new regulation in WNT signaling [14]. In a previous study, we sought to uncover new regulatory mechanisms in WNT signaling by performing forward genetic screens in HAP1-7TGP cells, a derivative of the haploid human cell line HAP1 harboring a fluorescent reporter of WNT signaling [15]. HAP1 cells are especially well suited for genetic screens due to the presence of a single allele of most genes in their near-haploid genome, which can be disrupted by mutagenesis to generate true genetic null cells [16]. We previously reported a comprehensive set of forward genetic screens designed to identify positive, negative and attenuating regulators of WNT/CTNNB1 signaling, as well as regulators of R-spondin (RSPO) signaling and suppressors of hyperactive WNT signaling induced by loss of distinct destruction complex components, including APC and CSNK1A1 [15]. These screens identified hits implicated at several levels of the pathway, from WNT and RSPO reception at the plasma membrane, to signal transduction in the cytosol, to transcriptional regulation in the nucleus. Comparative analyses of the screens enabled us to infer genetic interactions based on distinct patterns of hits identified by the different screens. The screens for suppressors of hyperactive signaling induced by loss of APC or CSNK1A1 suggested potential candidates for targeting oncogenic WNT signaling [15].

An unexpected outcome of the *APC* and *CSNK1A1* suppressor screens was that we observed only a partial overlap between significant hits in the two screens [15]. The phenotypic selection parameters used in both screens were the same, and the HAP1-7TGP cell lines used for the two screens were isogenic except for the mutations in *APC* or *CSNK1A1* we introduced by CRISPR/Cas9-mediated genome editing. Therefore, we expected that the hits identified in the two suppressor screens would be the same. After all, if APC and CSNK1A1 regulate WNT/CTNNB1 signaling through a single common function in the destruction complex phosphorylating CTNNB1, we assumed that hyperactivating the pathway by knocking out one or the other would be functionally equivalent, and the complement of downstream regulators would be shared. While there were indeed many common hits with high significance scores in both suppressor screens, including established downstream regulators of WNT/CTNNB1 signaling such as *CTNNB1* and *CREBBP*, there were also many hits unique to the *APC* suppressor or the *CSNK1A1* suppressor screen [15]. These results suggested that the hyperactive signaling state resulting from loss of these two destruction complex components was not equivalent. We hypothesized that the difference in potential downstream regulators in the two genetic backgrounds in which the screens were conducted – *APC* knock-out (KO) or *CSNK1A1* KO – could reflect additional roles of APC or CSNK1A1 in WNT/CTNNB1 signaling beyond their shared function regulating CTNNB1 abundance.

*HUWE1*, the gene encoding the eponymous E3 ubiquitin ligase, was the most striking example of a hit that was highly significant in the *CSNK1A1* suppressor but not the *APC* suppressor screen [15]. HUWE1 is a very large, 482 kilodalton (kDa) HECT domain E3 that has been implicated in many cellular processes, including transcriptional regulation, DNA replication and repair, cell cycle arrest, cell adhesion, cell migration, cell proliferation and differentiation, proteotoxic stress, ribosome biogenesis, mitochondrial maintenance, autophagy, apoptosis and WNT signaling [17–20]. *HUWE1* was the third most significant hit in the *CSNK1A1* suppressor screen, surpassed only by *CTNNB1* and *CREBBP*, which encode two of the main components of the TCF/LEF transcription complex and are therefore central players in the WNT pathway [15]. However, *HUWE1* was not a significant hit in the *APC* suppressor screen and it was not among the most significant hits in any of the screens performed in wild-type (WT) HAP1-7TGP cells, designed to identify positive regulators of WNT3A- and RSPO1-induced signaling [15]. These results suggested that HUWE1 might be involved in a regulatory mechanism that is most evident in CSNK1A1^KO^ cells (for brevity, HAP1-7TGP cell lines in which one or more genes were mutated will be referred to simply by the name of the protein(s) encoded by the mutated gene(s) followed by a superscript describing the mutation(s)). In follow-up studies, we had confirmed that HUWE1 loss reduced WNT target gene transcription – and to a smaller extent CTNNB1 abundance – in CSNK1A1^KO^ but not in APC^KO^ cells [15]. We had also shown that microinjection of *HUWE1* mRNA into *Xenopus laevis* embryos promoted body axis duplication, a hallmark of ectopic WNT signaling [15]. These experiments established a few biological contexts in which HUWE1 acts as a positive regulator of WNT/CTNNB1 signaling, but the underlying mechanism remained unclear and the reason why HUWE1 loss selectively reduced WNT/CTNNB1 signaling in CSNK1A1^KO^ cells remained unknown.

Here we extend our genetic analyses to show that HUWE1 enhances WNT/CTNNB1 signaling through two different mechanisms. First, HUWE1 reduces CTNNB1 phosphorylation and degradation by antagonizing the activity of a destruction complex containing GSK3A/GSK3B, APC and AXIN1, thereby increasing CTNNB1 abundance. Second, HUWE1 enhances WNT signaling through a mechanism that is independent from changes in CTNNB1 abundance.

## Results

### HUWE1 enhances WNT signaling in CSNK1A1^KO^ cells by antagonizing GSK3A/GSK3B-dependent phosphorylation of the CTNNB1 phosphodegron, thereby increasing CTNNB1 abundance

**Fig 1:**
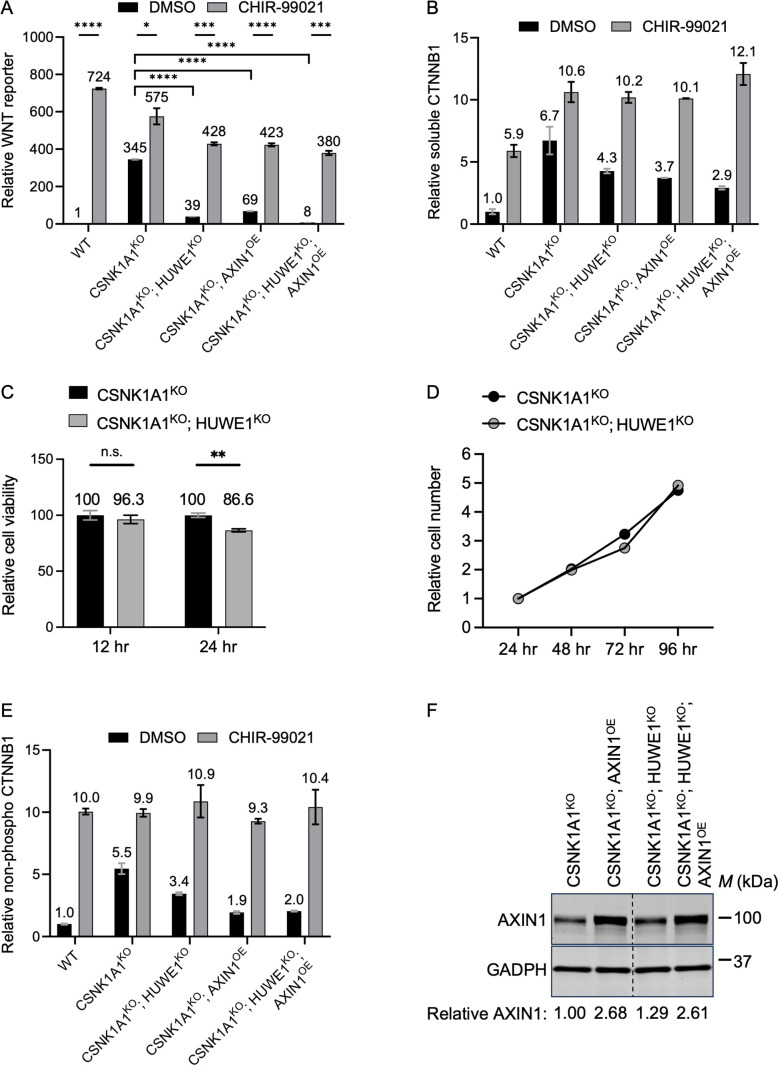
HUWE1 and AXIN1 reciprocally regulate WNT signaling by modulating GSK3A/GSK3B-dependent CTNNB1 phosphorylation and abundance. We note that the data for WT HAP-7TGP, CSNK1A1^KO^ and CSNK1A1^KO^; HUWE1^KO^ cells is discussed in the first section of the results, while the data for CSNK1A1^KO^; AXIN1^OE^ and CSNK1A1^KO^; HUWE1^KO^; AXIN1^OE^ cells is discussed in a later section of the results subtitled “HUWE1 enhances WNT signaling by antagonizing the destruction complex.” Cells were treated with DMSO vehicle or 10 µM of the GSK3A/GSK3B inhibitor CHIR-99021 for 48 hr where indicated. (A) WNT reporter activity (median EGFP fluorescence from 10,000 singlets was measured for triplicate wells and the average ± standard deviation (SD) of the three measurements is depicted), relative to WT HAP1-7TGP cells treated with DMSO. Significance was determined by unpaired t-test with Welch’s correction. (B) Soluble CTNNB1 abundance (CTNNB1 intensity normalized to total protein, average ± SD from duplicate immunoblots shown in S1A Fig) in membrane-free supernatants (MFS) of the indicated cell lines, relative to WT HAP1-7TGP cells treated with DMSO. (C) Cell viability assay. CellTiter-Glo luminescence (average ± SD from triplicate wells) measured at the indicated time points after seeding, relative to CSNK1A1^KO^ cells. Significance was determined by unpaired t-test with Welch’s correction. (D) Cell growth assay. Cell number (relative to the cell number 24 hr after seeding) at the indicated time points after seeding. (E) Non-phospho CTNNB1 (S33/S37/T41) abundance (non-phospho CTNNB1 intensity normalized to total protein, average ± SD from duplicate immunoblots shown in S1B Fig) in whole cell extracts (WCE) of the indicated cell lines, relative to WT HAP1-7TGP cells treated with DMSO. (F) Immunoblot analysis of total AXIN1 in WCE of the indicated cell lines used in A, B and E. The polyclonal cell populations overexpressing AXIN1 were generated as described in Materials and methods. AXIN1 abundance (AXIN1 intensity normalized to GAPDH intensity), relative to CSNK1A1^KO^ cells, is indicated below the blots.

We previously reported that HUWE1 loss in CSNK1A1^KO^ cells caused a substantial, 80–90% reduction in WNT reporter activity and endogenous WNT target gene expression that was accompanied by a smaller, 20–32% reduction in soluble CTNNB1 abundance [15] (soluble CTNNB1 measured in membrane-free supernatants (MFS; see Materials and methods) is a proxy for the signaling CTNNB1 pool because it excludes the more stable, plasma membrane-associated junctional CTNNB1 pool). We readily reproduced these results in the current study: HUWE1 loss in CSNK1A1^KO^ cells reduced WNT reporter activity by 89% and soluble CTNNB1 abundance by 36% (Figs 1A, 1B and S1A). These reductions were not likely to be caused by pleiotropic effects of HUWE1 loss, since knocking out *HUWE1* in CSNK1A1^KO^ cells resulted in only a small 4–15% reduction in cell viability (Fig 1C) and had no effect on cell growth (Fig 1D and S1 and S2 Movies). Therefore, we sought to determine the mechanism by which HUWE1 loss reduced CTNNB1 abundance.

The main mechanism that regulates CTNNB1 abundance is phosphorylation of the CTNNB1 phosphodegron by the destruction complex [5]. In CSNK1A1^KO^ cells we did not expect the phosphodegron to be phosphorylated by GSK3A/GSK3B at residues S33, S37 and T41 because phosphorylation of these residues generally requires the priming phosphorylation of residue S45 by CSNK1A1 [6,7]. Nevertheless, we tested whether the reduction in CTNNB1 abundance caused by HUWE1 loss in CSNK1A1^KO^ cells was due to increased phosphorylation of the CTNNB1 phosphodegron. For reasons discussed in S1 Text and S1B–S1E Fig, we evaluated the extent of CTNNB1 phosphodegron phosphorylation by immunoblotting for CTNNB1 that is *not* phosphorylated at residues S33, S37 and T41 (we refer to this species as non-phospho CTNNB1, but it is also known as active CTNNB1 [21]) in whole cell extracts (WCE; see Materials and methods) (Figs 1E and S1B). HUWE1 loss in CSNK1A1^KO^ cells reduced non-phospho CTNNB1 abundance by 37%, a reduction that correlated closely with the 36% reduction in soluble CTNNB1 abundance caused by HUWE1 loss in the same cell line (Figs 1B, 1E, S1A and S1B). This correlation suggested that the reduction in CTNNB1 abundance caused by HUWE1 loss was due to increased CTNNB1 phosphorylation at S33, S37 and T41, presumably mediated by GSK3A/GSK3B. If this were the case, inhibiting GSK3A/GSK3B should reverse the reduction in both soluble and non-phospho CTNNB1 abundance caused by HUWE1 loss. Treatment of CSNK1A1^KO^; HUWE1^KO^ cells with the GSK3A/GSK3B inhibitor CHIR-99021 indeed increased the abundance of soluble CTNNB1 by 2.4-fold and the abundance of non-phospho CTNNB1 by 3.2-fold (Figs 1B, 1E, S1A and S1B), entirely reversing the reductions caused by HUWE1 loss. Furthermore, GSK3A/GSK3B inhibition in CSNK1A1^KO^; HUWE1^KO^ cells increased WNT reporter activity by 10.9-fold, restoring signaling to a level comparable to that in DMSO vehicle-treated CSNK1A1^KO^ cells (Fig 1A). These results indicate that even in the absence of CSNK1A1, phosphorylation of residues S33, S37 and T41 by GSK3A/GSK3B can regulate CTNNB1 abundance, and that HUWE1 loss reduces CTNNB1 abundance and WNT signaling by promoting the phosphorylation of these residues.

Since HUWE1 loss in CSNK1A1^KO^ cells increased GSK3A/GSK3B-dependent phosphorylation of the CTNNB1 phosphodegron, we wondered whether in CSNK1A1^KO^ cells containing HUWE1, residues S33, S37 and T41 in the phosphodegron might be partially phosphorylated by GSK3A/GSK3B despite the absence of CSNK1A1. CSNK1A1^KO^ cells had a relatively high abundance of soluble and non-phospho CTNNB1, as well as high WNT reporter activity, compared to basal levels in unstimulated (DMSO vehicle-treated) WT HAP1-7TGP cells (Figs 1A, 1B, 1E, S1A and S1B). However, GSK3A/GSK3B inhibition with CHIR-99021 in CSNK1A1^KO^ cells increased the abundance of soluble CTNNB1 by 1.6-fold and the abundance of non-phospho CTNNB1 by 1.8-fold (Figs 1B, 1E, S1A and S1B). WNT reporter activity also increased 1.7-fold following treatment of CSNK1A1^KO^ cells with CHIR-99021 (Fig 1A). Therefore, in the absence of CSNK1A1, residual GSK3A/GSK3B-dependent phosphorylation of the CTNNB1 phosphodegron can still take place. This is presumably followed by ubiquitylation and proteasomal degradation of phosphorylated CTNNB1.

In summary, in CSNK1A1^KO^ cells, CTNNB1 is still phosphorylated by GSK3A/GSK3B at residues S33, S37 and S41 in the CTNNB1 phosphodegron, and the reduction in soluble CTNNB1 abundance caused by HUWE1 loss is due to increased GSK3A/GSK3B-dependent phosphorylation of these residues. We conclude that when present, HUWE1 antagonizes the GSK3A/GSK3B-dependent phosphorylation and ensuing degradation of CTNNB1, thereby increasing CTNNB1 abundance and promoting WNT signaling (Fig 6A).

### HUWE1 enhances WNT signaling through a mechanism independent of changes in CTNNB1 abundance

Our results raised the possibility that in CSNK1A1^KO^ cells, the large reduction in WNT signaling caused by HUWE1 loss was entirely due to the smaller but significant reduction in CTNNB1 abundance, since the transcriptional activity of transcriptional coactivators is not always proportional to their abundance. Alternatively, HUWE1 could conceivably regulate both CTNNB1 abundance and a distinct process that also modifies WNT signaling output. If HUWE1 loss disrupts both processes, it could result in a greater reduction in WNT target gene expression than in CTNNB1 abundance. Therefore, we sought to determine whether HUWE1 promotes WNT signaling through additional mechanisms distinct from regulation of CTNNB1 phosphodegron phosphorylation and the resulting changes in CTNNB1 abundance. Mutations in the CTNNB1 phosphodegron that prevent phosphorylation by CSNK1A1 and GSK3A/GSK3B render CTNNB1 insensitive to degradation mediated by the destruction complex [6,7,22]. We reasoned that introducing such mutations into the single *CTNNB1* allele of HAP1-7TGP cells would enable us to decouple control of CTNNB1 abundance from any other potential mechanism by which HUWE1 enhances WNT signaling.

**Fig 2:**
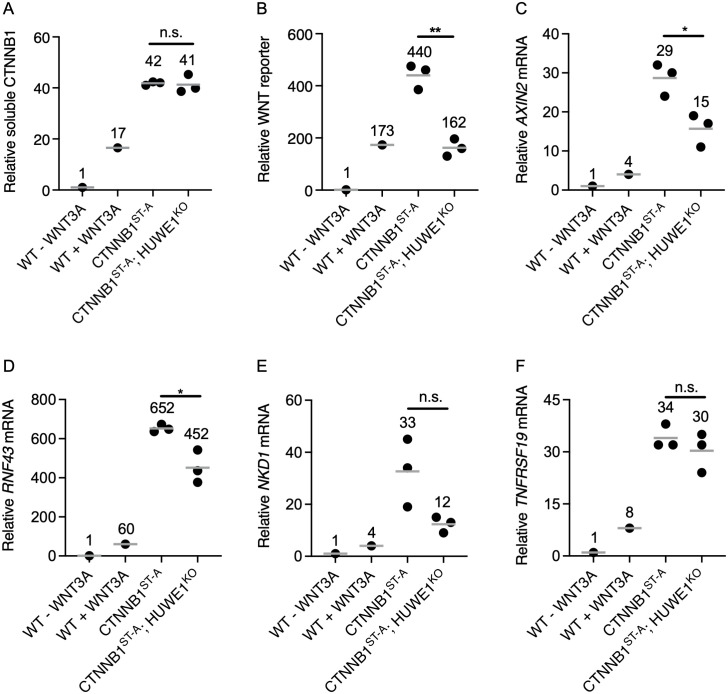
HUWE1 enhances WNT signaling through a mechanism independent of changes in CTNNB1 abundance. The same cell lines were used in A-F. Each circle represents a unique clonal cell line (determined by genotyping, S1 File). A single value for the parental WT HAP1-7TGP cell line, and the average value from 3 independent clonal cell lines for each of the other genotypes, all relative to untreated WT HAP1-7TGP cells, are indicated by a horizontal line and quantified above each group of circles. WT HAP1-7TGP cells were treated with 50% WNT3A CM for 24 hr where indicated. Significance was determined by unpaired t-test with Welch’s correction. (A) Relative soluble CTNNB1 abundance (CTNNB1 intensity normalized to total protein and GAPDH intensity, average from duplicate immunoblots shown in S2B Fig) in MFS of the indicated cell lines. (B) Relative WNT reporter activity (median EGFP fluorescence from 100,000 singlets). (C-F) Relative WNT target gene expression (average quantification of *AXIN2*, *RNF43*, *TNFRSF19*, or *NKD1* mRNA normalized to *HPRT1* mRNA, each measured in triplicate reactions).

We used CRISPR/Cas9-induced homology directed repair (HDR) to edit the codons encoding CSNK1A1 and GSK3A/GSK3B phosphorylation sites in the phosphodegron of the single endogenous *CTNNB1* locus in HAP1-7TGP cells. We introduced mutations encoding alanine (A) substitutions at residue S45, which is phosphorylated by CSNK1A1, and at residues T41 and S37, which are sequentially phosphorylated by GSK3A/GSK3B (S1 File and S2A Fig). We were unable to mutate S33, the third GSK3A/GSK3B target site. However, recognition of CTNNB1 by the SCF^βTrCP^ E3 ubiquitin ligase complex requires phosphorylation of both S37 and S33 [9,10] in the phosphodegron, and therefore the three mutations we introduced still prevented destruction complex-dependent CTNNB1 degradation, as we demonstrate below. We called the resulting HAP1-7TGP derivative cell line CTNNB1^ST-A^. The mutations in the *CTNNB1* locus of CTNNB1^ST-A^ cells indeed increased soluble CTNNB1 abundance 42-fold compared to unstimulated WT HAP1-7TGP cells (Figs 2A and S2B), and promoted constitutive WNT signaling as judged by WNT reporter activity and endogenous WNT target gene (*AXIN2* [23], *RNF43* [23], *NKD1* [24], *TNFRSF19* [25]) expression (Fig 2B–2F). Furthermore, soluble CTNNB1 abundance, WNT reporter activity and WNT target gene expression in CTNNB1^ST-A^ cells were substantially higher than in WT HAP1-7TGP cells treated with a near-saturating dose of WNT3A conditioned media (CM) (Figs 2A–2F and S2B). Stimulation of CTNNB1^ST-A^ cells with WNT3A CM did not significantly increase total CTNNB1 abundance or WNT target gene expression (S2C–S2E Fig). These results confirmed that the mutations we introduced into CTNNB1^ST-A^ cells rendered CTNNB1 insensitive to degradation by the destruction complex, and therefore abolished the control of CTNNB1 abundance by WNT ligands.

**Fig 3:**
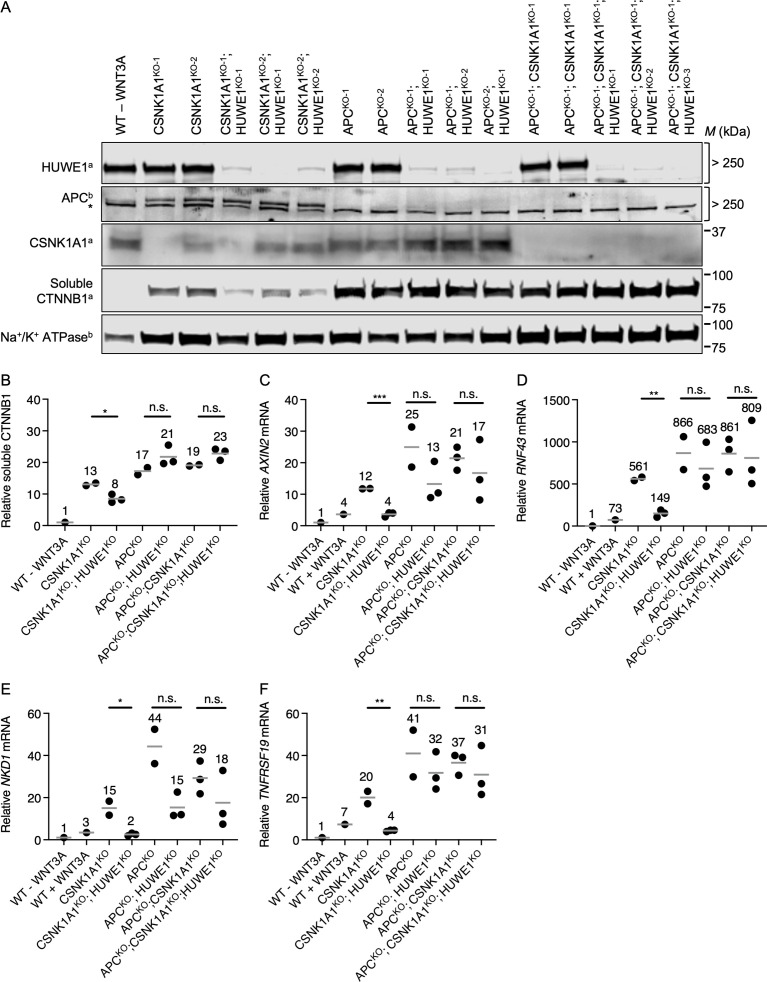
HUWE1 enhances WNT signaling through mechanisms mediated by APC. The same cell lines were used in A-F. WT HAP1-7TGP cells were treated with 50% WNT3A CM for 24 hr where indicated. (A) Immunoblot analysis of soluble proteins in MFS of the indicated clonal cell lines. We note that CSNK1A1^KO-2^ cells contain a loss-of-function mutation resulting in a 2-amino acid deletion (S1 File), and hence the protein product is still present. The “a” and “b” superscripts next to the protein names indicate which of two membranes the corresponding strips were cut from. The * in the APC blot indicates a non-specific band observed with the mouse anti-APC antibody. (B-F) Each circle represents a unique clonal cell line (determined by genotyping, S1 File). A single value for the parental WT HAP1-7TGP cell line, and the average value from 2-3 independent clonal cell lines for each of the other genotypes, all relative to untreated WT HAP1-7TGP cells, are indicated by a horizontal line and quantified above each group of circles. Significance was determined by unpaired t-test. (B) Relative soluble CTNNB1 abundance (CTNNB1 intensity normalized to total protein, average from duplicate immunoblots) in MFS of the indicated cell lines. (C–F) Relative WNT target gene expression (average quantification of *AXIN2*, *RNF43*, *TNFRSF19*, or *NKD1* mRNA normalized to *HPRT1* mRNA, each measured in triplicate reactions).

We then knocked out *HUWE1* in CTNNB1^ST-A^ cells (S1 File and S2B Fig) and measured the effect on soluble CTNNB1 abundance and WNT signaling. HUWE1 loss in multiple independent clonal cell lines (CTNNB1^ST-A^; HUWE1^KO^) did not affect soluble CTNNB1 abundance (Figs 2A and S2B), but significantly reduced WNT reporter activity (Figs 2B and S2F) and the expression of some WNT target genes (Fig 2C–2F). These results demonstrate that HUWE1 loss reduces WNT signaling in part through a mechanism independent from CTNNB1 phosphodegron phosphorylation and the resulting reduction in CTNNB1 abundance. We also note that the 49–63% reduction in WNT reporter activity, 45% reduction in *AXIN2* expression and 31% reduction in *RNF43* expression caused by HUWE1 loss in CTNNB1^ST-A^ cells (Figs 2B–2D and S2F) were smaller than the 89% reduction in WNT reporter activity, 67% reduction in *AXIN2* expression and 73% reduction in *RNF43* expression caused by HUWE1 loss in CSNK1A1^KO^ cells (Figs 1A, 3C and 3D). Since HUWE1 loss caused a 36% reduction in soluble CTNNB1 abundance in CSNK1A1^KO^ cells (Figs 1B and S1A) but no corresponding reduction in CTNNB1^ST-A^ cells (Figs 2A and S2B), the larger overall reduction in WNT signaling caused by HUWE1 loss in CSNK1A1^KO^ cells may be explained by the contribution from two distinct mechanisms in CSNK1A1^KO^ cells but only one of these mechanisms in CTNNB1^ST-A^ cells.

In summary, we distinguished two mechanisms whereby HUWE1 loss reduces WNT signaling. In CSNK1A1^KO^ cells containing WT CTNNB1, HUWE1 loss caused a moderate reduction in CTNNB1 abundance and a comparable increase in GSK3A/GSK3B-dependent phosphorylation of the CTNNB1 phosphodegron, as well as a much larger GSK3A/GSK3B-dependent reduction in WNT reporter activity (Fig 1). In CTNNB1^ST-A^ cells containing WT CSNK1A1 but a mutated CTNNB1 phosphodegron, HUWE1 loss did not alter CTNNB1 abundance but still caused a significant reduction in WNT reporter activity and WNT target gene expression (Fig 2). We conclude that HUWE1 enhances WNT signaling through two distinct mechanisms, one that antagonizes GSK3A/B-dependent CTNNB1 phosphorylation and degradation, thereby increasing CTNNB1 abundance (Fig 1), and another that is independent of changes in CTNNB1 abundance (Fig 2). These results are summarized schematically in Fig 6A.

### HUWE1 enhances WNT signaling through mechanisms mediated by APC

Having defined two mechanisms whereby HUWE1 enhances WNT signaling, a GSK3A/GSK3B-dependent mechanism that regulates CTNNB1 phosphorylation and abundance, and another mechanism that is independent of changes in CTNNB1 abundance, we wondered whether these mechanisms are also mediated by other destruction complex components. HUWE1 was one of the most significant hits in a *CSNK1A1* suppressor screen but was not a significant hit in an *APC* suppressor screen in HAP1-7TGP cells [15]. Consistent with the results of these screens, HUWE1 loss substantially reduced WNT reporter activity in CSNK1A1^KO^ cells but did not affect WNT reporter activity in APC^KO^ cells [15]. Based on these results, we hypothesized that APC may be required to mediate the effects of HUWE1 on WNT signaling, potentially explaining why HUWE1 loss had no effect in the absence of APC. If APC is required for the reduction in WNT signaling caused by HUWE1 loss in CSNK1A1^KO^ cells, then eliminating APC function in CSNK1A1^KO^; HUWE1^KO^ cells, like inhibiting GSK3A/GSK3B activity (Fig 1), should reverse said reduction. We are not aware of any pharmacological inhibitors that we could use to acutely inhibit APC. We were also unable to knock out *APC* in CSNK1A1^KO^; HUWE1^KO^ cells, as we found that knocking out additional genes by CRISPR/Cas9-mediated genome editing in this cell line yielded very few viable clones. Instead, to test whether APC mediates the effects of HUWE1 on WNT signaling, we first made cell lines lacking both APC and CSNK1A1, and then tested the effects of HUWE1 loss in these cells, comparing them to cells lacking CSNK1A1 alone.

*CSNK1A1* single KO clonal HAP1-7TGP cell lines were generated and characterized previously [15] (CSNK1A1^KO-1^ and CSNK1A1^KO-2^; we note that CSNK1A1^KO-2^ is a loss-of-function allele containing a two-amino acid deletion). We generated two new *APC* single KO clonal HAP1-7TGP cell lines (APC^KO-1^ and APC^KO-2^) as well as two new *APC* and *CSNK1A1* double KO clonal HAP1-7TGP cell lines (APC^KO-1^; CSNK1A1^KO-1^ and APC^KO-1^; CSNK1A1^KO-2^) using CRISPR/Cas9-mediated genome editing. We validated these cell lines by sequencing each targeted locus (S1 File) and by immunoblot analysis (Fig 3A). CSNK1A1^KO^, APC^KO^ and APC^KO^; CSNK1A1^KO^ cells all exhibited elevated soluble CTNNB1 abundance several-fold higher than unstimulated WT HAP1-7TGP cells (Fig 3A and 3B). All these clonal cell lines exhibited constitutive expression of WNT target genes several-fold higher than the level of gene expression in unstimulated WT HAP1-7TGP cells and in WT HAP1-7TGP cells stimulated with a near-saturating dose of WNT3A CM (Fig 3C–3F). Consistent with our results demonstrating that in CSNK1A1^KO^ cells residual phosphorylation of the CTNNB1 phosphodegron by GSK3A/GSK3B results in some CTNNB1 degradation (Fig 1), both soluble CTNNB1 abundance and WNT target gene expression were higher in APC^KO^ and APC^KO^; CSNK1A1^KO^ cells than in CSNK1A1^KO^ cells (Fig 3A–3F).

We then knocked out *HUWE1* in CSNK1A1^KO^, APC^KO^ and APC^KO^; CSNK1A1^KO^ cells to generate three CSNK1A1^KO^; HUWE1^KO^, three APC^KO^; HUWE1^KO^ and three APC^KO^; CSNK1A1^KO^; HUWE1^KO^ clonal cell lines, which we validated by sequencing the targeted *HUWE1* locus (S1 File) and by immunoblot analysis (Fig 3A). HUWE1 loss in CSNK1A1^KO^ cells substantially reduced the expression of all WNT target genes tested (Fig 3C–3F) and, to a lesser extent, soluble CTNNB1 abundance (Fig 3A and 3B). In contrast, HUWE1 loss in APC^KO^ cells resulted in a variable but not statistically significant reduction in WNT target gene expression (Fig 3C–3F) and did not reduce soluble CTNNB1 abundance (Fig 3A and 3B), consistent with our previous finding that HUWE1 loss in APC^KO^ cells had no effect on WNT reporter activity [15]. Finally, HUWE1 loss in APC^KO^; CSNK1A1^KO^ cells yielded equivalent results to those in APC^KO^ cells, showing a variable but not statistically significant reduction in WNT target gene expression (Fig 3C–3F), and no reduction in soluble CTNNB1 abundance (Fig 3A and 3B). Even if HUWE1 loss in APC^KO^ and/or APC^KO^; CSNK1A1^KO^ cells caused a modest reduction in WNT target gene expression that we could not measure reliably, this reduction was much smaller than in CSNK1A1^KO^ cells. These results indicate that, like GSK3A/GSK3B inhibition, APC loss precludes or significantly diminishes the reduction in WNT target gene expression and CTNNB1 abundance caused by HUWE1 loss in CSNK1A1^KO^ cells. We conclude that APC mediates the effects of HUWE1 on WNT signaling (Fig 6A).

### HUWE1 enhances WNT signaling through mechanisms mediated by a subset of destruction complex components including APC, AXIN1 and GSK3A or GSK3B

We extended the same logic as for APC (Fig 3) to test the role of every core component of the destruction complex in mediating the functions of HUWE1 in WNT signaling. We first knocked out components of the destruction complex individually or in certain combinations (Table 1) so we could then test the effects of HUWE1 loss on WNT signaling in each of these mutant genetic backgrounds. While we had already established the role of GSK3A/GSK3B and APC in mediating HUWE1 functions (Figs 1 and 3), we included them in our analysis to confirm those results and, in the case of GSK3A and GSK3B, to test their roles individually. We used CRISPR/Cas9-mediated genome editing to generate HAP1-7TGP clonal cell lines lacking the desired destruction complex components (Table 1). We confirmed that each targeted genomic locus had been successfully mutated (S1 File) and that the encoded protein had been eliminated (S3A Fig). We noticed that knocking out certain destruction complex components changed the electrophoretic mobility and/or abundance of others, especially of APC, AXIN1 and AXIN2 (S3A Fig). This may be due to destabilization of one destruction complex scaffold in the absence of its binding partner, or due to changes in the extent of phosphorylation of these destruction complex scaffolds by the destruction complex kinases, a process that can also regulate their stability [26].

**Table 1:** HUWE1 enhances WNT signaling through mechanisms mediated by a subset of destruction complex components including APC, AXIN1 and GSK3A or GSK3B. Summary of effects of CRISPRi-mediated *HUWE1* KD on the aggregate expression of four endogenous WNT target genes (Fig 4E; see figure legend for more details). For the genotypes and treatments indicated in green, both HUWE1 sgRNAs used resulted in a significant reduction in aggregate WNT target gene expression relative to the SCR sgRNA control.

Genotype and treatment	Aggregate WNT target gene expression for HUWE1 sgRNA1/ sgRNA2 as % of SCR sgRNA control	Significance of change in aggregate WNT target gene expression for HUWE1 sgRNA1/ sgRNA2 relative to SCR sgRNA control
WT - WNT3A	101/101	n.s./n.s.
WT + WNT3A	68/72	n.s./n.s.
CSNK1A1^KO^	40/55	**/**
APC^KO^	94/79	n.s./n.s.
APC^KO^; CSNK1A1^KO^	81/91	n.s./n.s.
AXIN1^KO^; AXIN2^KO^	88/95	n.s./n.s.
CSNK1A1^KO^; AXIN1^KO^; AXIN2^KO^	111/96	n.s./n.s.
CSNK1A1^KO^; AXIN1^KO^	83/107	n.s./n.s.
CSNK1A1^KO^; AXIN2^KO^	42/49	**/**
GSK3A^KO^; GSK3B^KO^	88/88	n.s./n.s.
CSNK1A1^KO^; GSK3A^KO^; GSK3B^KO^	119/164	*/n.s.
CSNK1A1^KO^; GSK3A^KO^	46/60	*/*
CSNK1A1^KO^; GSK3B^KO^	37/ 43	**/**

To test the effects of HUWE1 loss in each of these 12 cell lines (Table 1 and S3A Fig), we adopted a different experimental strategy: CRISPR interference (CRISPRi)-mediated knock-down (KD) [27]. For reasons discussed in S2 Text, in this case CRISPRi was preferable to clonal analysis because it enabled us to measure the outcome of *HUWE1* KD in polyclonal cell populations rather than *HUWE1* KO in multiple individual clonal cell lines. We used a lentivirus to deliver the CRISPRi machinery together with sgRNAs targeting *HUWE1* in the various cell lines we had generated lacking destruction complex components (Table 1 and S3A Fig). Based on immunoblot measurements (S4A and S4B Fig), lentiviral delivery of either of two different sgRNAs targeting *HUWE1* (HUWE1 sgRNA1 or sgRNA2) followed by antibiotic selection of transduced cells resulted in a consistent 59–95% reduction of HUWE1 compared to a control, scrambled (SCR) sgRNA. We refer to polyclonal cell populations in which we knocked down *HUWE1* using CRISPRi as HUWE1^KD^, in contrast to HUWE1^KO^ clonal cell lines in which we knocked out *HUWE1* using CRISPR/Cas9-mediated genome editing.

**Fig 4:**
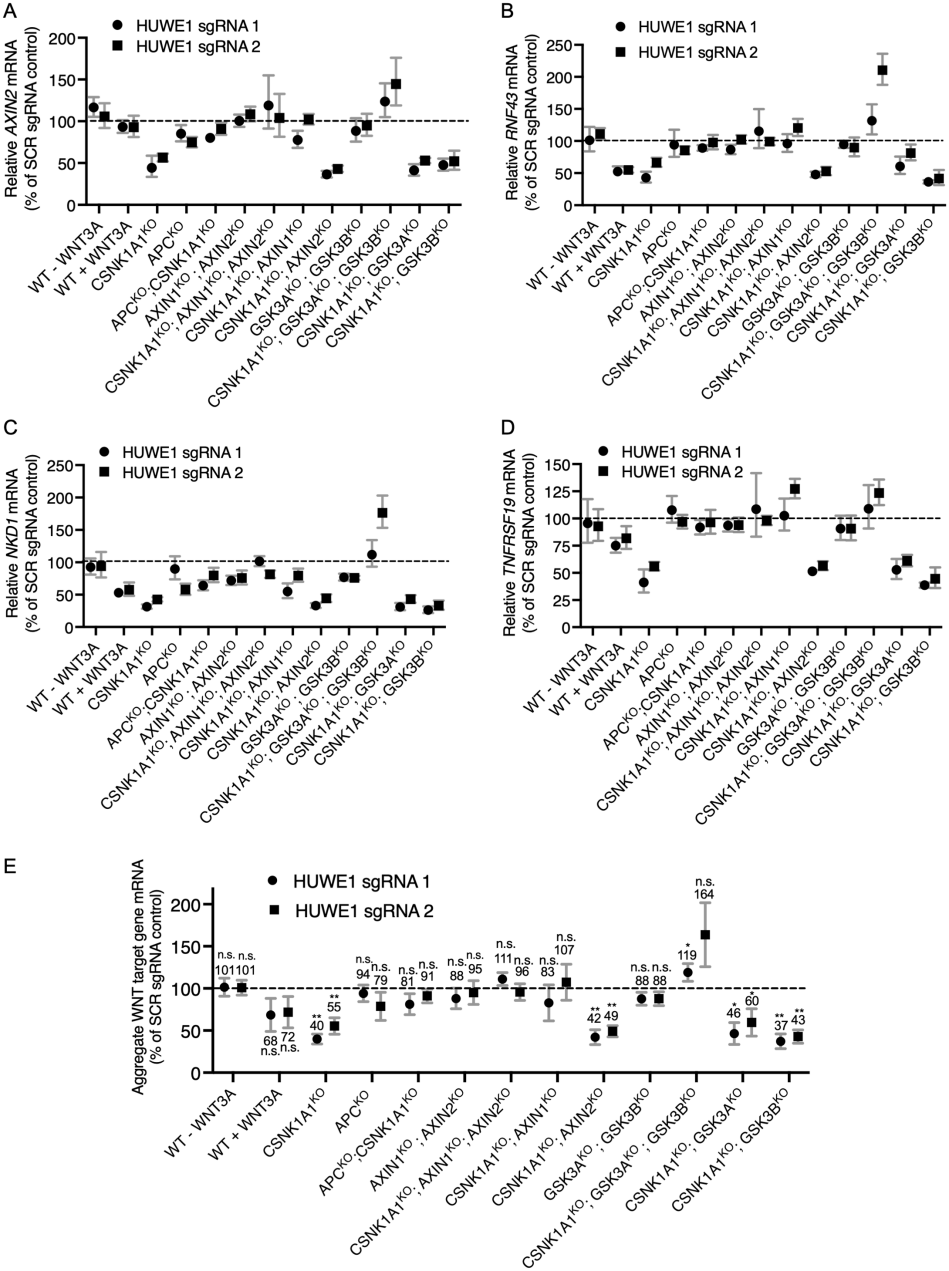
HUWE1 enhances WNT signaling through mechanisms mediated by a subset of destruction complex components including APC, AXIN1 and GSK3A or GSK3B. The same cell lines were used in A-E. WT HAP1-7TGP cells were treated with 50% WNT3A CM for 24 hr where indicated. (A–D) Endogenous WNT target gene expression (average ± SD *AXIN2*, *RNF43*, *TNFRSF19*, or *NKD1* mRNA normalized to *HPRT1* mRNA, each measured in triplicate reactions) in polyclonal cell populations targeted for CRISPRi-mediated *HUWE1* KD with HUWE1 sgRNA 1 or 2, reported as percentage of WNT target gene expression in polyclonal cell populations targeted with SCR sgRNA control. (E) Aggregate endogenous WNT target gene expression (average ± SD of normalized *AXIN2*, *RNF43*, *TNFRSF19* and *NKD1* mRNA, quantified in A-D) in polyclonal cell populations targeted with HUWE1 sgRNA 1 or 2, reported as percentage of aggregate WNT target gene expression in polyclonal cell populations targeted with SCR sgRNA control. Significance relative to SCR was determined by paired t-test.

To validate the CRISPRi KD strategy, we tested whether knocking down *HUWE1* in CSNK1A1^KO^, APC^KO^, and APC^KO^; CSNK1A1^KO^ cell populations produced equivalent results to those we had observed when we knocked out *HUWE1* and conducted clonal analysis in these same cell lines (Fig 3). Consistent with our clonal analysis, *HUWE1* KD in CSNK1A1^KO^ cells significantly reduced the expression of four endogenous WNT target genes compared to CSNK1A1^KO^ cells transduced with SCR sgRNA (Fig 4A–4E and Table 1). However, this reduction was smaller than that caused by HUWE1 loss in CSNK1A1^KO^; HUWE1^KO^ cells (Fig 3C–3F), presumably owing to some residual HUWE1 protein present in CSNK1A1^KO^; HUWE1^KD^ cells (S4A and S4B Fig). Also consistent with our clonal analysis, *HUWE1* KD in APC^KO^ and in APC^KO^; CSNK1A1^KO^ cells did not cause a reproducible, statistically significant reduction in WNT target gene expression (Fig 4A–4E and Table 1). These results validate CRISPRi-mediated *HUWE1* KD in polyclonal cell populations as a reliable alternative to the more laborious clonal analysis of multiple individual HUWE1^KO^ clonal cell lines.

We then asked whether AXIN1 and/or AXIN2 mediate the functions of HUWE1 in WNT signaling. In WT HAP1-7TGP cells, AXIN1 and AXIN2 are functionally redundant in their capacity to suppress WNT signaling by regulating CTNNB1 abundance as scaffolds in the destruction complex: eliminating either AXIN1 or AXIN2 has no effect on WNT reporter activity, whereas eliminating both promotes constitutive pathway activation [15]. We initially assumed that a possible role of *AXIN1* and *AXIN2* in mediating the functions of HUWE1 may also be redundant, so we tested whether *HUWE1* KD reduced constitutive WNT signaling in AXIN1^KO^; AXIN2^KO^ double KO cells (S1 File and S3A Fig). *HUWE1* KD in AXIN1^KO^; AXIN2^KO^ cells (S4A and S4B Fig) did not reduce WNT target gene expression (Fig 4A–4E and Table 1), suggesting that AXIN1, AXIN2 or both mediate the reduction in WNT signaling caused by HUWE1 loss, similarly to what we had observed for APC. We also tested whether the combined loss of AXIN1 and AXIN2 eliminated the reduction in WNT signaling caused by *HUWE1* KD in CSNK1A1^KO^ cells. Indeed, knocking down *HUWE1* in CSNK1A1^KO^; AXIN1^KO^; AXIN2^KO^ cells did not reduce WNT target gene expression as it did in CSNK1A1^KO^ cells (Fig 4A–4E and Table 1). These results confirmed that in CSNK1A1^KO^ cells, AXIN1, AXIN2 or both mediate the functions of HUWE1 in WNT signaling.

While AXIN1 and AXIN2 are redundant in their capacity to suppress WNT signaling in WT HAP1-7TGP cells [15], it was conceivable that they may not be redundant in mediating the functions of HUWE1. To test for individual contributions of *AXIN1* or *AXIN2*, we knocked each of them out individually in CSNK1A1^KO^ cells (S1 File and S3A Fig) and then knocked down *HUWE1* (S4A and S4B Fig). AXIN1 loss in CSNK1A1^KO^ cells eliminated the reduction in WNT signaling caused by *HUWE1* KD, but AXIN2 loss did not (Fig 4A–4E and Table 1). These results suggest that, in contrast to its redundant function with AXIN2 in suppressing WNT signaling in WT HAP1-7TGP cells [15], AXIN1 plays a unique role in mediating HUWE1-dependent effects on WNT signaling that is not redundant with AXIN2.

Given these results, we wondered whether GSK3A and GSK3B are redundant in mediating the functions of HUWE1 in WNT signaling. To answer this question, we did an equivalent series of experiments as the one we did to determine the individual roles of AXIN1 and AXIN2 in mediating HUWE1 function. Like AXIN1 and AXIN2, GSK3A and GSK3B are functionally redundant in their capacity to suppress WNT signaling in WT HAP1-7TGP cells: eliminating either GSK3A or GSK3B has no effect on WNT reporter activity, whereas eliminating both promotes constitutive pathway activation (S1 File, and S3B and S3C Fig). *HUWE1* KD in GSK3A^KO^; GSK3B^KO^ cells (S4A and S4B Fig) did not reduce WNT target gene expression (Fig 4A–4E and Table 1), suggesting that GSK3A, GSK3B or both mediate the reduction in WNT signaling caused by HUWE1 loss. Next, we tested whether the combined loss of GSK3A and GSK3B eliminated the reduction in WNT signaling caused by *HUWE1* KD in CSNK1A1^KO^ cells. Knocking down *HUWE1* in CSNK1A1^KO^; GSK3A^KO^; GSK3B^KO^ cells (S4A and S4B Fig) did not reduce – in fact increased – WNT target gene expression (Fig 4A–4E and Table 1). These results confirmed our previous conclusion (Fig 1) that GSK3A, GSK3B or both mediate the reduction in WNT signaling caused by HUWE1 loss in CSNK1A1^KO^ cells. However, unlike their combined loss, loss of GSK3A or GSK3B individually in CSNK1A1^KO^ cells did not eliminate the reduction in WNT signaling caused by *HUWE1* KD (Fig 4A–4E and Table 1). We conclude that the role of GSK3A and GSK3B in mediating HUWE1-dependent effects on WNT signaling is redundant, similarly to their role suppressing WNT signaling in WT HAP1-7TGP cells (S3C Fig). Therefore, only the combined loss of GSK3A and GSK3B eliminates the reduction in WNT signaling caused by *HUWE1* KD in CSNK1A1^KO^ cells (Fig 4A–4E and Table 1).

We also knocked down *HUWE1* in WT HAP1-7TGP cells containing an intact destruction complex (S4A and S4B Fig), in which we had previously reported that *HUWE1* KO did not cause a significant reduction in WNT reporter activity or *AXIN2* expression induced by a near-saturating dose of WNT3A [15]. In agreement with those results, *HUWE1* KD in WT HAP1-7TGP cells did not reduce WNT3A-induced expression of *AXIN2* (Fig 4A). However, *HUWE1* KD in WT HAP1-7TGP cells reduced the expression of other WNT target genes, including *RNF43*, *NKD1* and *TNFRSF19*, but to a smaller extent than in CSNK1A1^KO^ cells (Fig 4B–4D). Given these differences between distinct WNT target genes, we also conducted clonal analysis to compare the expression of all four genes between three WT HAP1-7TGP and three HUWE1^KO^ clonal cell lines we had isolated previously (S1 File) [15]. We obtained similar results as with CRISPRi-mediated *HUWE1* KD: *HUWE1* KO did not reduce *AXIN2* expression, but significantly reduced *RNF43*, *NKD1* and *TNFRSF19* expression following treatment with a near-saturating dose of WNT3A (S4C–S4F Fig). We cannot explain why HUWE1 loss failed to reduce the expression of *AXIN2* but reduced the expression of other WNT target genes selectively in WT HAP1-7TGP cells. However, together with our analysis in CTNNB1^ST-A^ cells (Fig 2), these results demonstrate that the contribution of HUWE1 to WNT signaling is not limited to cells lacking CSNK1A1.

We considered the possibility that the distinct outcomes of knocking down *HUWE1* in the various genetic backgrounds we tested (Table 1) could be due to differences in the steady state abundance of HUWE1 caused by loss of some destruction complex components but not others, rather than due to other effects of distinct destruction complex components in mediating HUWE1 functions. Standard immunoblot analysis did not reveal obvious differences in steady state HUWE1 abundance among the various genetic backgrounds in which we knocked down *HUWE1* (S3A Fig). We confirmed this result by quantitative dot blot analysis (see Materials and methods) and did not detect significant differences in HUWE1 abundance among the different genetic backgrounds (S3D Fig).

In conclusion, a subset of destruction complex components, including APC, AXIN1 and GSK3A or GSK3B, but not CSNK1A1 or AXIN2, mediates the reduction in WNT signaling caused by HUWE1 loss. Since HUWE1 enhances WNT signaling by increasing CTNNB1 abundance (Fig 1) and through another mechanism independent of changes in CTNNB1 abundance (Fig 2), we conclude that a destruction complex containing APC, AXIN1 and GSK3A/GSK3B must mediate the effects of HUWE1 on one or both mechanisms (Fig 6A).

### HUWE1 enhances WNT signaling by antagonizing the destruction complex

The results presented so far are consistent with the following hypothesis (Fig 6A): 1. in CSNK1A1^KO^ cells, APC, AXIN1 and GSK3A/GSK3B are part of a residual destruction complex that can partially suppress WNT signaling by phosphorylating the CTNNB1 phosphodegron and targeting CTNNB1 for degradation; 2. HUWE1 enhances WNT signaling by antagonizing CTNNB1 phosphorylation and degradation mediated by this destruction complex, and through another mechanism independent of changes in CTNNB1 abundance. Whether the second mechanism is also mediated by the destruction complex remains unclear.

If HUWE1 enhances WNT signaling in CSNK1A1^KO^ cells by antagonizing the activity of a residual destruction complex, we reasoned that ectopically increasing destruction complex activity should counteract the function of HUWE1 and reduce WNT signaling. In other words, increasing destruction complex activity even in the absence of CSNK1A1 should phenocopy the effects of HUWE1 loss. Destruction complex activity can be increased by stabilizing destruction complex scaffolds. For example, tankyrase inhibitors reduce WNT signaling by stabilizing AXIN1 and AXIN2 [28]. However, tankyrase has other substrates in addition to AXIN1 and AXIN2 [29], so we pursued an overexpression strategy to ectopically increase destruction complex activity. AXIN1 is a limiting component of the destruction complex in some contexts [30] and we found that loss of AXIN1, but not AXIN2, in CSNK1A1^KO^ cells selectively abolished the reduction in WNT signaling caused by HUWE1 loss (Fig 4). Therefore, we hypothesized that overexpressing AXIN1 in CSNK1A1^KO^ cells might increase destruction complex activity, promote CTNNB1 phosphorylation and degradation, and reduce WNT signaling, thereby phenocopying the effects of HUWE1 loss. Furthermore, overexpressing AXIN1 in CSNK1A1^KO^; HUWE1^KO^ cells – in which we postulate that WNT signaling is reduced because HUWE1 no longer antagonizes the destruction complex – might further reduce signaling by promoting ectopic destruction complex activity.

To test these hypotheses, we stably overexpressed human AXIN1 in CSNK1A1^KO^ and in CSNK1A1^KO^; HUWE1^KO^ cells through lentiviral delivery of *AXIN1* cDNA followed by antibiotic selection. We obtained polyclonal cell populations (CSNK1A1^KO^; AXIN1^OE^ and CSNK1A1^KO^; HUWE1^KO^; AXIN1^OE^, respectively) in which AXIN1 abundance was at least 2-fold higher than that in the respective parental cell lines (Fig 1F). AXIN1 overexpression in CSNK1A1^KO^ cells reduced WNT reporter activity by 80%, which was comparable to the 89% reduction caused by HUWE1 loss in CSNK1A1^KO^ cells (Fig 1A). AXIN1 overexpression combined with HUWE1 loss in CSNK1A1^KO^ cells reduced WNT reporter activity by 98%, nearly down to the basal level of unstimulated WT HAP1-7TGP cells (Fig 1A). Therefore, AXIN1 overexpression in CSNK1A1^KO^ cells phenocopied HUWE1 loss, and AXIN1 overexpression in CSNK1A1^KO^; HUWE1^KO^ cells synergized with HUWE1 loss to reduce WNT signaling. We conclude that HUWE1 and AXIN1 exert opposing effects on WNT signaling.

To test whether the reductions in WNT signaling caused by AXIN1 overexpression and by HUWE1 loss in CSNK1A1^KO^ cells were due to the same underlying mechanisms – increased CTNNB1 phosphorylation leading to CTNNB1 degradation – we measured the abundance of soluble, non-phospho and total CTNNB1 in CSNK1A1^KO^; AXIN1^OE^ and CSNK1A1^KO^; HUWE1^KO^; AXIN1^OE^ cells, as we had done in WT HAP1-7TGP, CSNK1A1^KO^ and CSNK1A1^KO^; HUWE1^KO^ cells (Figs 1B, 1E, S1A, S1B and S1E). AXIN1 overexpression in CSNK1A1^KO^ cells reduced soluble CTNNB1 abundance by 45% and non-phospho CTNNB1 abundance by 64% (Figs 1B, 1E, S1A and S1B). These reductions were greater than the respective 36% and 37% reductions caused by HUWE1 loss in CSNK1A1^KO^ cells (Figs 1B, 1E, S1A and S1B). AXIN1 overexpression combined with HUWE1 loss in CSNK1A1^KO^ cells reduced soluble CTNNB1 abundance by 57% and non-phospho CTNNB1 by 62% (Figs 1B, 1E, S1A and S1B). These results indicate that HUWE1 and AXIN1 have opposing functions regulating a common mechanism: AXIN1 promotes CTNNB1 phosphorylation and degradation, while HUWE1 antagonizes both.

If HUWE1 and AXIN1 exert opposing effects on WNT signaling by regulating the same process – CTNNB1 phosphorylation and degradation mediated by GSK3A/GSK3B – then GSK3A/GSK3B inhibition should reverse the effects of AXIN1 overexpression in CSNK1A1^KO^ cells, just as it reversed the effects of HUWE1 loss (Figs 1A, 1B, 1E, S1A, S1B and S1E). Therefore, we tested whether the reductions in WNT reporter activity, soluble CTNNB1 abundance and CTNNB1 phosphodegron phosphorylation caused by AXIN1 overexpression alone or combined with HUWE1 loss were dependent on GSK3A/GSK3B activity. Treatment of CSNK1A1^KO^; AXIN1^OE^ cells with the GSK3A/GSK3B inhibitor CHIR-99021 reversed the effects of AXIN1 overexpression, increasing WNT reporter activity as well as soluble and non-phospho CTNNB1 abundance to levels higher than those measured in DMSO vehicle treated-CSNK1A1^KO^ cells, and comparable to the levels measured in CHIR-99021-treated CSNK1A1^KO^ cells (Figs 1A, 1B, 1E, S1A and S1B). GSK3A/GSK3B inhibition with CHIR-99021 also reversed the synergistic reduction in WNT reporter activity, as well as the reductions in soluble and non-phospho CTNNB1 abundance, caused by combined AXIN1 overexpression and HUWE1 loss in CSNK1A1^KO^ cells (Figs 1A, 1B, 1E, S1A and S1B). These results demonstrate that in CSNK1A1^KO^ cells, HUWE1 enhances and AXIN1 inhibits WNT signaling by opposing GSK3A/GSK3B-dependent CTNNB1 phosphorylation and degradation.

Altogether, our results support the hypothesis that AXIN1, acting as a scaffold in the destruction complex, promotes GSK3A/GSK3B-dependent CTNNB1 phosphorylation and degradation even in the absence of CSNK1A1. HUWE1 enhances WNT signaling by antagonizing the activity of this residual destruction complex (Fig 6A).

### Regulation of WNT signaling by HUWE1 requires its ubiquitin ligase activity

HUWE1 is a very large (482 kDa) E3 with many protein-protein interaction domains in addition to its catalytic HECT domain [31]. HUWE1 loss could conceivably reduce WNT signaling by eliminating such protein-protein interactions, including scaffolding functions, rather than by preventing the ubiquitylation of a protein substrate. Therefore, it was important to determine whether the ubiquitin ligase activity of HUWE1 is required for its functions enhancing WNT signaling. To this end, we generated CSNK1A1^KO^ cells containing only catalytically dead HUWE1 protein and tested its effects on WNT signaling. HECT domain E3s form a covalent intermediate between a catalytic cysteine (C) residue in the HECT domain and ubiquitin before ubiquitin is transferred to the substrate [32]. We used CRISPR-mediated base editing [33] to engineer the single endogenous *HUWE1* locus in CSNK1A1^KO^ cells, introducing a point mutation that replaced the catalytic C4341 residue in the HECT domain with arginine (R). A catalytically dead HUWE1 protein containing a similar C4341S mutation was used previously to study HUWE1 function [31]. We isolated three independent clonal cell lines, CSNK1A1^KO^; HUWE1^C4341R-1^, CSNK1A1^KO^; HUWE1^C4341R-2^ and CSNK1A1^KO^; HUWE1^C4341R-3^, in which we confirmed by sequencing that the intended point mutation had been introduced (S1 File).

**Fig 5:**
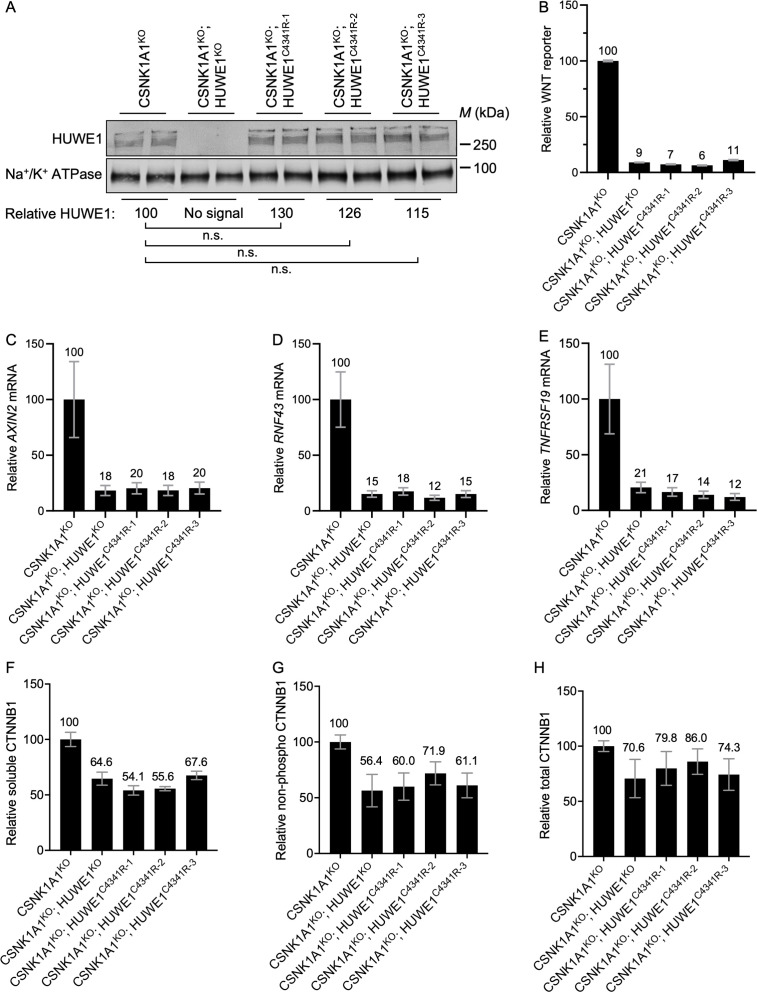
Regulation of WNT signaling by HUWE1 requires its ubiquitin ligase activity. (A) Immunoblot analysis of total HUWE1 protein in WCE of the cell lines used in B-H. Replicate samples were loaded in adjacent lanes. Average HUWE1 abundance (HUWE1 intensity normalized to Na^+^/K^+^ ATPase intensity), relative to CSNK1A1^KO^ cells, is indicated below the blots. Significance was determined by paired t-test. (B) WNT reporter activity (median ± standard error of the median (SEM) EGFP fluorescence from 37,000-50,000 cells) for one CSNK1A1^KO^; HUWE1^KO^ clonal cell line and three independent, catalytically dead CSNK1A1^KO^; HUWE1^C4341R^ clonal cell lines, relative to CSNK1A1^KO^ cells. (C–E) WNT target gene expression (average ± SD *AXIN2*, *RNF43* or *TNFSRF19* mRNA normalized to *HPRT1* mRNA, each measured in triplicate reactions) for the indicated clonal cell lines, relative to CSNK1A1^KO^ cells. (F) Soluble CTNNB1 abundance (CTNNB1 intensity normalized to GAPDH, average ± SD from duplicate immunoblots shown in S5D Fig) in MFS of the indicated cell lines, relative to CSNK1A1^KO^ cells. (G) Non-phospho CTNNB1 (S33/S37/T41) abundance (non-phospho CTNNB1 intensity normalized to GAPDH, average ± SD from duplicate immunoblots shown in S5E Fig) in WCE of the indicated cell lines, relative to CSNK1A1^KO^ cells. (H) Total CTNNB1 abundance (CTNNB1 intensity normalized to GAPDH, average ± SD from duplicate immunoblots shown in S5E Fig) in WCE of the indicated cell lines, relative to CSNK1A1^KO^ cells.

We used structural prediction analysis to assess whether the C4341R mutation is likely to abolish the catalytic activity of HUWE1 by eliminating the reactive C residue rather than by disrupting the local fold of the HECT domain, which could also affect protein-protein interactions. We first compared a structural model of the WT HUWE1 HECT domain generated by AlphaFold 3 to the previously solved crystal structure of this domain (S5A Fig). The two structures were nearly superimposable, confirming that the structural model generated by AlphaFold 3 was accurate. We then generated a structural model of the mutant HUWE1^C4341R^ HECT domain using AlphaFold 3 and compared it to the crystal structure (S5B Fig) or to the AlphaFold 3-generated structural model of the WT HUWE1 HECT domain (S5C Fig). Both comparisons confirmed that the mutant HUWE1^C4341R^ and WT HUWE1 HECT domains were nearly superimposable, except for the R side chain in the mutant protein, which extended out into the open catalytic cleft and therefore was unlikely to disrupt the folding of the HECT domain. We also tested whether the C4341R mutation perturbed the overall stability of HUWE1 by immunoblot analysis. Unfolded proteins are often degraded, but the C4341R point mutation did not reduce the amount of HUWE1 in WCE from the three CSNK1A1^KO^; HUWE1^C4341R^ clonal cell lines compared to CSNK1A1^KO^ cells (Fig 5A). In contrast, no HUWE1 protein was detected in CSNK1A1^KO^; HUWE1^KO^ cells (Fig 5A).

We compared the effects of eliminating the catalytic activity of HUWE1 versus knocking out *HUWE1* on WNT signaling and CTNNB1 abundance. All three CSNK1A1^KO^; HUWE1^C4341R^ clonal cell lines exhibited a substantial 89–94% reduction in WNT reporter activity (Fig 5B) and an 80–88% reduction in the expression of three endogenous WNT target genes (Fig 5C–5E), equivalent to the 91% reduction in WNT reporter activity (Fig 5B) and 79–85% reduction in endogenous WNT target gene expression we observed in CSNK1A1^KO^; HUWE1^KO^ cells. Furthermore, in the three CSNK1A1^KO^; HUWE1^C4341R^ clonal cell lines, soluble CTNNB1 abundance was reduced by 32–46%, non-phospho CTNNB1 by 28–40% and total CTNNB1 by 14–26% (Figs 5F–5H, S5D and S5E). These reductions were comparable to the respective 35%, 44% and 29% reductions measured in CSNK1A1^KO^; HUWE1^KO^ cells (Figs 5F–5H, S5D and S5E). We conclude that the ubiquitin ligase activity of HUWE1 is required for its functions enhancing WNT signaling.

## Discussion

**Fig 6:**
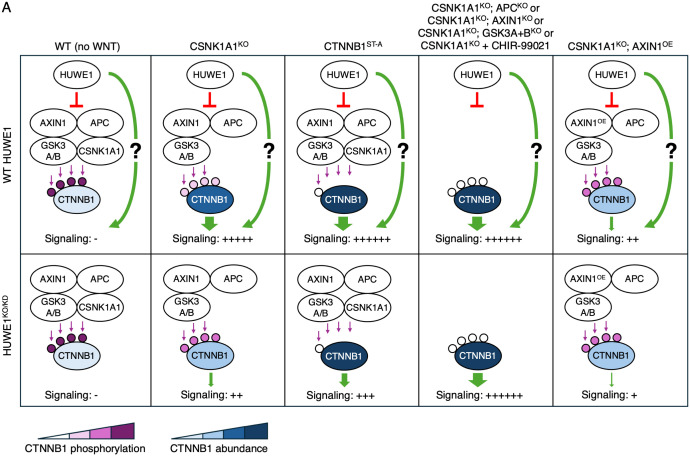
WNT signaling regulation by HUWE1. (A) Schematic diagram with scenarios illustrating many of the genotypes and/or treatments tested in this study, highlighting two distinct mechanisms whereby HUWE1 enhances WNT/CTNNB1 signaling: 1. by antagonizing CTNNB1 phosphorylation and degradation mediated by the destruction complex, and 2. through another mechanism independent of changes in CTNNB1 abundance. The circles on CTNNB1 represent (from left to right) the three GSK3A/B and the single CSNK1A1 phosphorylation sites in the phosphodegron, and the purple arrows indicate phosphorylation of these sites by the respective kinases. The graded purple scale represents the extent of CTNNB1 phosphorylation, and the graded blue scale represents CTNNB1 abundance. Relative WNT signaling levels in each scenario (indicated by green arrows of different widths below CTNNB1, and by –/ + signs) are intended to be approximations. See Results and Discussion sections for more detailed explanations of each scenario.

In this study we probed the mechanisms by which HUWE1, a HECT domain E3, enhances WNT/CTNNB1 signaling [15]. We demonstrate that HUWE1 enhances WNT/CTNNB1 signaling through two distinct mechanisms: by antagonizing destruction complex-mediated CTNNB1 phosphorylation and degradation, and through another mechanism independent of changes in CTNNB1 abundance (Fig 6A). These results are significant for two main reasons. First, they reveal a new mechanism whereby HUWE1 controls CTNNB1 phosphorylation and degradation, the main regulated step in WNT/CTNNB1 signaling. Second, by controlling another step in the WNT/CTNNB1 pathway distinct from CTNNB1 abundance, HUWE1 contributes a new layer of regulation superimposed on the core signaling module. Importantly, the coordinated regulation of CTNNB1 abundance and an independent signaling step by HUWE1 would be an efficient way to control multiple processes that determine WNT signaling output. This may enable sensitive and robust activation of the pathway.

We found that in CSNK1A1^KO^ cells, GSK3A/GSK3B still phosphorylated a fraction of CTNNB1 at residues S33, S37 and T41 in the phosphodegron, which reduced CTNNB1 abundance (Fig 1). HUWE1 enhances signaling by antagonizing destruction complex-dependent phosphorylation of these residues, since HUWE1 loss in CSNK1A1^KO^ cells increased CTNNB1 phosphorylation and reduced both CTNNB1 abundance and WNT signaling activity (Fig 1). However, the reduction in CTNNB1 abundance caused by HUWE1 loss was smaller than the reduction in WNT target gene expression (Fig 1), raising the possibility that HUWE1 also enhances WNT/CTNNB1 signaling through another mechanism. Indeed, in CTNNB1^ST-A^ cells containing mutations in the CTNNB1 phosphodegron that render CTNNB1 abundance insensitive to regulation by WNT ligands and the destruction complex, HUWE1 enhances WNT target gene expression through a mechanism independent of changes in CTNNB1 abundance (Fig 2). Furthermore, regulation of WNT/CTNNB1 signaling by HUWE1 is mediated by a subset of destruction complex components, including APC, AXIN1 and GSK3A or GSK3B, but excluding CSNK1A1 and AXIN2 (Figs 1, 3 and 4). HUWE1 promotes WNT signaling by antagonizing the activity of this destruction complex (Fig 1). The ubiquitin ligase activity of HUWE1 is required to enhance WNT signaling (Fig 5), suggesting that a ubiquitylated substrate or substrates of HUWE1 mediate its functions in WNT signaling.

One of the mechanisms whereby HUWE1 enhances WNT/CTNNB1 signaling is by antagonizing phosphorylation of the CTNNB1 phosphodegron by the destruction complex, thereby increasing CTNNB1 abundance, but surprisingly this happens in the absence of CSNK1A1 (Fig 6A). These results demonstrate that in HAP1 cells, CSNK1A1 is not absolutely required for GSK3A/GSK3B-dependent phosphorylation of residues S33, S37 and T41 in the CTNNB1 phosphodegron, either because GSK3A/GSK3B can phosphorylate these residues without the priming phosphorylation of S45 by CSNK1A1, or because other kinases phosphorylate S45 in the absence of CSNK1A1. While priming of S45 by CSNK1A1 is generally considered a requirement for phosphorylation of S33, S37 and T41 by GSK3A/GSK3B [6,7], some reports suggest it is not [34,35].

We also show there is another mechanism whereby HUWE1 enhances WNT/CTNNB1 signaling that is independent of changes in CTNNB1 abundance (Fig 6A). HUWE1 could potentially regulate CTNNB1 subcellular localization or its interactions with the TCF/LEF transcription complex, or it could regulate other downstream steps in the pathway. Elucidating this second mechanism and whether it is also mediated by a subset of destruction complex components, like HUWE1-dependent regulation of CTNNB1 abundance, will be crucial to understand the full scope of how HUWE1 regulates WNT signaling.

Intriguingly, only a subset of destruction complex components, including APC, AXIN1 and GSK3A or GSK3B, but not CSNK1A1 or AXIN2, mediate the function of HUWE1 in WNT signaling (Figs 1, 3 and 4). We were surprised to find that AXIN1 was required to mediate the effects of HUWE1 but AXIN2 was not. In WT HAP1-7TGP cells, AXIN1 and AXIN2 are redundant in their capacity to suppress WNT signaling: eliminating either AXIN1 or AXIN2 has no effect on WNT reporter activity, whereas eliminating both results in constitutive pathway activation [15]. Yet, in CSNK1A1^KO^ cells, only AXIN1 loss eliminated the reduction in WNT target gene expression caused by *HUWE1* KD (Fig 4 and Table 1). These results suggest that AXIN1 and AXIN2 are not redundant in their capacity to mediate the effects of HUWE1, at least in the absence of CSNK1A1. This finding is unexpected given that mouse AXIN1 and AXIN2 proteins have been reported to be functionally equivalent *in vivo* [36], and will require further investigation.

The ubiquitin ligase activity of HUWE1 is required to promote WNT signaling in CSNK1A1^KO^ cells (Fig 5). What are the relevant ubiquitylated HUWE1 substrates, and how do they regulate WNT signaling? Does HUWE1-dependent ubiquitylation target putative substrates for proteasomal degradation or does it regulate their subcellular localization or activity? Since a subset of destruction complex components mediates the effects of HUWE1 on WNT signaling, is the abundance or activity of a destruction complex component regulated by HUWE1-dependent ubiquitylation or are there other ubiquitylated substrates that indirectly impinge on destruction complex abundance, subcellular localization or activity? Identification of the relevant HUWE1 substrates should help answer these questions.

We demonstrate that HUWE1 loss reduces WNT signaling in cells containing mutations in some WNT pathway components but not in others (Figs 1–5). These results raise the possibility of targeting the signaling mechanisms by which HUWE1 enhances WNT signaling selectively in tumors harboring mutations in specific WNT pathway components. Eliminating or reducing the activity of HUWE1 itself, which in turn reduces WNT/CTNNB1 signaling in WT HAP1-7TGP, CSNK1A1^KO^ and CTNNB1^ST-A^ cells, is unlikely to be a viable therapeutic strategy due to the multiple roles of HUWE1 on cell physiology, including tumor suppressor functions [37]. However, if the relevant ubiquitylated target of HUWE1 is identified, there may be other ways to phenocopy the effects of HUWE1 loss on WNT signaling more selectively. Phenocopying the effects of HUWE1 loss may not be effective in tumors driven by APC truncations, given that in APC^KO^ cells HUWE1 loss does not reduce WNT signaling due to the role of APC itself in mediating the effects of HUWE1. However, in tumors containing activating mutations in CTNNB1 like those engineered into our CTNNB1^ST-A^ cell line, or mutations in the ZNRF3 or RNF43 tumor suppressors, all of which result in hyperactive WNT signaling in the presence of a functional destruction complex, phenocopying the effects of HUWE1 loss may reduce WNT signaling enough to provide a therapeutic benefit.

Previous reports have implicated HUWE1 as a *negative* regulator of WNT signaling [38–41]. HUWE1 has been reported to polyubiquitylate DVL and prevent DVL multimerization [38], which is required to form a functional signalosome and transduce WNT signals [42,43]. HUWE1 has also been reported to ubiquitylate CTNNB1 and promote CTNNB1 degradation [39]. Based on both reported mechanisms, HUWE1 loss would be expected to promote rather than reduce WNT signaling. This is the opposite of what we find in WT HAP1-7TGP, CSNK1A1^KO^ and CTNNB1^ST-A^ cells, in which HUWE1 is a *positive* regulator of the pathway: eliminating HUWE1 or its catalytic activity in these cells substantially reduces WNT/CTNNB1 signaling (Figs 1–5). In fact, in CSNK1A1^KO^ cells, HUWE1 loss or mutation of its catalytic C residue reduces CTNNB1 abundance (Figs 1, 3 and 5), in direct contradiction to one of the previously reported mechanisms [39]. Therefore, we do not think that DVL or CTNNB1 are the relevant ubiquitylated substrates that mediate the effects of HUWE1 on WNT signaling in HAP1 cells. These disparate results could reflect differences in experimental systems, since the previous reports primarily studied HUWE1 in *C. elegans* and HEK293T cells [38,39], while the experiments presented in the current study were conducted in HAP1 cells. Identifying the substrate of HUWE1 that mediates its role as a positive regulator of WNT/CTNNB1 signaling in HAP1 cells should help explain these differences.

We recognize that all the experiments presented in the Results section of this manuscript were conducted in HAP1-7TGP cells or derivatives thereof, which could raise concerns about the generality of our conclusions. We also studied HUWE1 in other cell lines commonly used in WNT signaling research, but our attempts to knock out *HUWE1* yielded only partial KOs. We targeted *HUWE1* by CRISPR/Cas9-mediated genome editing in HEK293T-7TG and HEK293T-7TG CSNK1A1^KO^ cells (see Materials and methods). Out of 113 independent clonal cell lines in which we identified mutations in all *HUWE1* alleles, at least one allele had been repaired in frame to encode WT HUWE1 protein (S6A and S6B Fig). This is probably because HUWE1 is a common essential gene as per DEPMAP classification (https://depmap.org/portal/gene/HUWE1?tab=overview), so complete loss of HUWE1 may be lethal in HEK293T cells. However, we have previously shown that microinjection of *HUWE1* mRNA into *Xenopus* embryos results in body axis duplication [15], consistent with a more general role of HUWE1 as a positive regulator of WNT signaling beyond HAP1 cells.

Given its many known substrates and the plethora of cellular processes regulated by HUWE1 [17–20], questions could be raised about the specificity of the effects on WNT signaling caused by HUWE1 loss. However, our results provide strong evidence that the downregulation of WNT signaling caused by HUWE1 loss was not due to non-specific or pleiotropic effects. First, we observed consistent reductions in WNT signaling when we knocked out or knocked down *HUWE1* in 6 different genetic backgrounds – WT HAP1-7TGP, CSNK1A1^KO^, CTNNB1^ST-A^, CSNK1A1^KO^; AXIN2^KO^, CSNK1A1^KO^; GSK3A^KO^ and CSNK1A1^KO^; GSK3B^KO^ cells (Figs 1–5 and Table 1) – but importantly, HUWE1 loss did not reduce WNT signaling in 7 other closely related genetic backgrounds – APC^KO^, APC^KO^; CSNK1A1^KO^, AXIN1^KO^; AXIN2^KO^, CSNK1A1^KO^; AXIN1^KO^; AXIN2^KO^, CSNK1A1^KO^; AXIN1^KO^, GSK3A^KO^; GSK3B^KO^ and CSNK1A1^KO^; GSK3A^KO^; GSK3B^KO^ cells (Figs 3, 4 and Table 1). It seems highly unlikely that non-specific or pleiotropic effects would be so strongly dependent on genetic background. Second, downregulation of WNT signaling caused by HUWE1 loss could be reversed completely by a relatively short and specific pharmacological treatment with the GSK3A/GSK3B inhibitor CHIR-99021 (Fig 1). This would not be expected unless any non-specific or pleiotropic effects of HUWE1 loss were also mediated by GSK3A/GSK3B. Finally, HUWE1 loss had only minor effects on cell viability and did not affect cell growth (Fig 1). Altogether, these results make it very unlikely that the reduction in WNT signaling caused by HUWE1 loss is non-specific or due to pleiotropic effects on unrelated cellular functions that indirectly impinge on WNT signaling.

Our study also highlights the remarkable potential of HAP1 haploid cells to dissect complex genetic networks in a cell line of human origin [16] through a combination of genome-wide forward genetic screens, loss-of-function and site-directed mutagenesis analyses, and genetic interaction analyses. Despite great advances in CRISPR/Cas-based genome editing technologies during the last decade [44], it remains challenging to knock out two or more alleles of multiple genes and to introduce targeted homozygous point mutations at scale in diploid primary cells, stem cells, and polyploid immortalized cell lines. We could readily do both in HAP1 cells because they have a single allele of most genes. This enabled us to conduct loss-of-function genetic analyses in multiple genetic backgrounds by comparing several *HUWE1* KO and control WT clonal cell lines to obtain highly quantitative phenotypic data that confirmed and extended the results of our initial genetic screens (Figs 3 and S4). Using CRISPR/Cas9-mediated HDR, we generated a CTNNB1 variant in which we mutated three key phosphorylation sites in the phosphodegron at the single endogenous *CTNNB1* locus, and in a second round of CRISPR/Cas9-mediated genome editing we generated multiple *HUWE1* KO and WT cell lines to demonstrate that regulation of WNT signaling by HUWE1 has a component that is independent of changes in CTNNB1 abundance (Fig 2). Using CRISPR-mediated base editing, we generated three clonal cell lines containing a point mutation in the catalytic C residue of HUWE1 at the single endogenous *HUWE1* locus, which enabled us to demonstrate that the ubiquitin ligase activity of HUWE1 is required for its function in WNT signaling (Fig 5). Finally, we generated single, double, and triple KO mutants for all components of the destruction complex, alone and in certain combinations (11 distinct mutant genetic backgrounds in total) (S3A Fig and Table 1). Combined with a CRISPRi strategy, this enabled us to carry out an extensive genetic interaction analysis and demonstrate that positive regulation of WNT signaling by HUWE1 is mediated by a subset of destruction complex components (Fig 4 and Table 1). These kinds of genetic analyses would have been practically impossible to conduct in any diploid or polyploid human cell line. We hope this study will inspire other researchers to take advantage of haploid human cell lines, of which there are now many available [45,46], to unravel other signaling pathways or biological processes in similar ways.

HUWE1 has emerged as an important E3 with many cellular functions [17–20]. Here we show another role for HUWE1 regulating WNT/CTNNB1 signaling. Regulation of CTNNB1 abundance by the destruction complex is the central step in WNT/CTNNB1 signaling. Our discovery that HUWE1 enhances WNT signaling by antagonizing destruction complex-dependent CTNNB1 phosphorylation, thereby increasing CTNNB1 abundance, demonstrates that this crucial step in WNT/CTNNB1 signaling is subject to more nuanced regulation than previously thought. The second mechanism by which HUWE1 enhances WNT signaling independently of changes in CTNNB1 abundance is an intriguing additional layer of regulation that remains to be elucidated. Both mechanisms provide new insights into WNT signaling and ubiquitin biology, bridging two research fields that already have many intimate connections.

## Materials and methods

The following Materials and methods relevant to this manuscript have been described previously [15]: cell lines and growth conditions, preparation of WNT3A conditioned media and construction of the HAP1-7TGP WNT reporter haploid cell line.

### Tissue culture media and growth conditions

Complete growth medium (CGM) 1 contains Dulbecco’s Modified Eagles Medium (DMEM) with High Glucose, without L-Glutamine and Sodium Pyruvate (GE Healthcare Life Sciences Cat. # SH30081.01); 1X GlutaMAX-I (Thermo Fisher Scientific Cat. # 35050079); 1X MEM Non-Essential Amino Acids (Thermo Fisher Scientific Cat. # 11140050); 1 mM Sodium Pyruvate (Thermo Fisher Scientific Cat. # 11360070); 40 Units/ml Penicillin, 40 mg/ml Streptomycin (Thermo Fisher Scientific Cat. # 15140122); 10% Fetal Bovine Serum (FBS).

CGM 2 contains Iscove’s Modified Dulbecco’s Medium (IMDM) with L-glutamine, with HEPES, without Alpha-Thioglycerol (GE Healthcare Life Sciences Cat. # SH30228.01); 1X GlutaMAX-I; 40 Units/ml Penicillin, 40 mg/ml Streptomycin; 10% FBS.

Cells were grown in a humidified incubator set to 37 °C and 5% CO_2_.

### Plasmids

pX330-U6-Chimeric_BB-CBh-hSpCas9 (pX330) (Addgene plasmid # 42230) was a gift from Feng Zhang; pCMV_ABEmax_P2A_GFP (Addgene plasmid # 112101) was a gift from David Liu; MLM3636 (Addgene plasmid # 43860) was a gift from Keith Joung; Lenti-(BB)-EF1a-KRAB-dCas9-P2A-BlastR (Addgene plasmid # 118154) was a gift from Jorge Ferrer; LentiCRISPRv2-mCherry (Addgene plasmid # 99154) was a gift from Agata Smogorzewska; pMDLg/pRRE (Addgene plasmid # 12251), pRSV-Rev (Addgene plasmid # 12253) and pMD2.G (Addgene plasmid # 12259) were a gift from Didier Trono; pCS2-YFP was a gift from Henry Ho; pmCherry was a gift from Jan Carette; pX458-mCherry was generated as described previously [47].

The following plasmids were purchased: pLenti6.2/V5-DEST (Thermo Fisher Scientific Cat. # V36820); pENTR2B (Thermo Fisher Scientific Cat. # A10463); MGC Human AXIN1 Sequence-verified cDNA (Clone ID 5809104) (Horizon Cat. # MHS6278-202833071).

To generate pCMV_ABEmax_P2A_mCherry, mCherry was amplified by PCR from plasmid pmCherry using primers pCMV_ABEmax_P2A_mCherry_Fw (5’-GAA GCA GGC TGG AGA CGT GGA GGA GAA CCC TGG ACC TAT GGT GAG CAA GGG CGA GGA-3’) and pCMV_ABEmax_P2A_mCherry_Rv (5’-CAG ACT TGT ACA GCT CGT CCA TGC CG-3’), designed to include BsmBI and BsrGI restriction sites, respectively. The PCR product was digested with BsmBI and BsrGI and ligated into pCMV_ABEmax_P2A_GFP digested with the same enzymes to replace GFP with mCherry.

To generate pLenti6.2-V5-EXP-N-TERM-S-FLAG-N-hAXIN1, human AXIN1 was amplified by PCR from MGC Human AXIN1 Sequence-verified cDNA (Clone ID 5809104) using forward primer pENTR2B_SalI_S-FLAG-N_hAXIN1_pcr_fw (5’-GCG CCG GAA CCA ATT CAG TCG ACC CTG CAG GAT GGA TTA CAA GGA CGA CGA TGA CAA GGG CGG CCG CAT GAA TAT CCA AGA GCA GGG TTT CCC CTT GGA CC-3’), containing an N-terminal SalI restriction site followed by a FLAG tag sequence flanked by SbfI and NotI restriction sites, and reverse primer pENTR2B_XhoI_hAXIN1_pcr_rv (5’-AAA GCT GGG TCT AGA TAT CTC GAG TCA GTC CAC CTT CTC CAC TTT GCC GAT GA-3’), containing a C-terminal XhoI restriction site. The product was digested with SalI and XhoI, and subcloned into pENTR2B digested with the same enzymes. One clone was sequenced completely and subcloned into pLenti6.2/V5-DEST using the Gateway LR Clonase II Enzyme mix.

All constructs were confirmed by sequencing.

### Antibodies

Primary antibodies: purified mouse anti-β-catenin (Clone 14/Beta-Catenin) (1:1000, BD Biosciences Cat. # 610154), rabbit mAb anti-non-phospho (active) β-catenin (Ser33-37-Thr41) (D13A1) (1:1000, Cell Signaling Technology Cat. # 8814), mouse anti-GAPDH (1:4000, Santa Cruz Biotechnology, Cat. # sc-47724), recombinant rabbit anti-Sodium Potassium (Na^+^/K^+^) ATPase [EP1845Y] (1:4000, Abcam Cat. # ab76020), rabbit anti-Lasu1/Ureb1 (HUWE1) (1:1000, Bethyl Laboratories Cat. # A300-486A), rabbit mAb anti-AXIN1 (C76H11) (1:1000, Cell Signaling Technology Cat. # 2087), rabbit mAb anti-AXIN2 (76G6) (1:500, Cell Signaling Technology Cat. # 2151), rabbit mAb anti-GSK-3α/β (D75D3) (1:2000, Cell Signaling Technology Cat. # 5676), mouse anti-APC (NT, clone Ali 12.28) (1:1000, Millipore Sigma, Cat. # MAB3785), rabbit anti-APC (1:1000, Biorbyt Cat. # orb213564), mouse anti-CSNK1A1 (1:250, Santa Cruz Biotechnology, Cat. # sc-74582).

Secondary antibodies: IRDye 800CW donkey anti-mouse IgG (H+L) (1:10000, Li-Cor Cat. # 926-32212), IRDye 680RD donkey anti-rabbit IgG (H+L) (1:10000, Li-Cor Cat. # 925-68073), peroxidase AffiniPure donkey anti-goat IgG (H+L) (1:5000, Jackson ImmunoResearch Laboratories Cat. # 705-035-003), peroxidase AffiniPure goat anti-rabbit IgG (H+L) (1:10000, Jackson ImmunoResearch Laboratories Cat. # 111-035-003), peroxidase AffiniPure donkey anti-mouse IgG (H+L) (1:5000, Jackson ImmunoResearch Laboratories Cat. # 715-035-150), goat anti-mouse IgG (H+L) HRP conjugate (1:10000, Bio-Rad Cat. # 1706516).

Primary and secondary antibodies used for detection with the Li-Cor Odyssey imaging system were diluted in a 1 to 1 mixture of Odyssey Intercept Blocking Buffer (Li-Cor Cat. # 927–40000) and TBST (Tris buffered saline (TBS) + 0.1% Tween-20), and those used for detection by chemiluminescence were diluted in TBST + 5% skim milk. All primary antibody incubations were done overnight at 4°C, and secondary antibody incubations were done for 1 hr at room temperature (RT).

### Construction of mutant HAP1-7TGP cell lines by CRISPR/Cas9-mediated genome editing

Oligonucleotides encoding single guide RNAs (sgRNAs) (S2 File) were selected from a published library [48] or designed using either of two online CRISPR design tools [49,50], and they were cloned into either pX330 or pX458-mCherry according to a published protocol [51].

Clonal HAP1-7TGP cell lines were established by transient transfection with either pX330 or pX458-mCherry containing the sgRNA followed by single cell sorting as follows. A transfection mix was prepared by diluting 450 ng of pX330 and 50 ng of pmCherry (used as a cotransfection marker for FACS sorting) or 500 ng of pX458-mCherry in 48 µl Opti-MEM I, adding 2 µl of X-tremeGENE HP and incubating for 20 min at RT. HAP1-7TGP cells or derivatives thereof were reverse-transfected in a well of a 24-well plate by overlaying 0.5 ml of CGM 2 (without antibiotics) containing 6 x 10^5^ cells over the 50 µl of transfection mix. Cells were passaged to a 10 cm dish ~24 hr post-transfection, using 150 µl of Trypsin-EDTA (0.25%) (Thermo Fisher Scientific Cat. # 25200056) to detach them (reverse-transfection of HAP1 cells caused unusually high adherence, hence the higher trypsin concentration). Three to four days post-transfection, single transfected (mCherry^+^) cells were sorted into 96-well plates containing 200 µl of CGM 2 per well and grown undisturbed for 16–18 days. Single colonies were passaged to 24-well plates, and a small number of cells was reserved for genotyping.

For genotyping, genomic DNA was extracted by adding 4 volumes of QuickExtract DNA Extraction Solution (Epicentre Cat. # QE09050) to the cells. Extracts were incubated 10 min at 65°C, 3 min at 98°C, and 5 µl were used as input for PCR amplification of the genomic locus containing the sgRNA target site in 15 µl reactions containing 1X LongAmp Taq reaction buffer, 300 mM of each dNTP, 400 nM of each of the flanking primers indicated in S2 File (designed using the Primer-BLAST online tool from NCBI) and 0.1 units/µl of LongAmp Taq DNA polymerase (NEB Cat. # M0323L). Amplification of the genomic locus containing the sgRNA target site was confirmed by analysis of the PCR products on a 1% agarose gel and the presence of desired mutations was confirmed by sequencing the amplicons using the primers indicated in S2 File. Given that most engineered cell lines remained haploid, sequencing results were usually unequivocal. Sequencing results for all the clonal cell lines used in the study is presented in S1 File, and for selected clonal cell lines, immunoblot analysis confirmed the absence of the protein products.

Whenever possible, multiple independent mutant cells lines, often generated using two different sgRNAs (see S1 File), were expanded and used for further characterization. For some of the comparisons between WT and mutant cells, multiple individual cell lines confirmed by sequencing to be WT at the sgRNA target site were also expanded and used as controls (we refer to this experimental scheme in which we compared multiple mutant and WT clonal cell lines as clonal analysis). To generate double and triple mutant cell lines, a single clonal cell line with the first desired mutation was used in a subsequent round of transfection with either pX330 or pX458-mCherry containing the second and, if applicable, third sgRNAs. Alternatively, WT HAP1–7TGP cells were directly transfected with a combination of pX330 or pX458-mCherry constructs targeting two genes simultaneously.

### Construction of CTNNB1^ST-A^ cell line by CRISPR/Cas9-mediated HDR

Oligonucleotides encoding sgRNAs complementary to exon 3 of *CTNNB1* (S2 File) were designed using either of two online CRISPR design tools [49,50] and cloned into pX458-mCherry using a published protocol [51].

Clonal CTNNB1^ST-A^ cell lines were established by transient transfection of HAP1-7TGP cells with pX458-mCherry containing the sgRNA, and a single stranded oligonucleotide (ssODN) donor template encoding the desired mutations, called CTNNB1 (ST-A mutant) donor (5’-ATT TGA TGG AGT TGG ACA TGG CCA TGG AAC CAG ACA GAA AAG CGG CTG TTA GTC ACT GGC AGC AAC AGT CTT ACC TGG ACG CTG GAA TCC ATG CTG GTG CCA CTG CCA CAG CTC CTG CTC TGA GTG GTA AAG GCA ATC CTG AGG AAG AGG ATG TGG ATA CCT CCC AAG TCC TGT ATG AGT GGG AAC AGG GAT TTT CTC AG-3’). A transfection mix was prepared by diluting 500 ng pX458-mCherry-CTNNB1-Ex3-sgRNA and 500 ng (8 pmol) ssODN in 48 µl Opti-MEM I. 2 µl of X-tremeGENE HP were added, and the mix was vortexed and incubated for 20 min at RT. The 50 µl mix was placed in an empty well of a 24-well plate and 0.5 ml of CGM 2 containing 6 x 10^5^ cells was seeded onto the mix. The cells were passaged the following day to a 10 cm dish and grown for 3 additional days. Single cells exhibiting high EGFP fluorescence from the 7TGP WNT reporter, presumably due to successful mutagenesis of the CTNNB1 phosphodegron, were sorted, expanded, and genotyped as described above. A single clonal cell line containing point mutations in three of the four targeted sites in the phosphodegron (S2A Fig and S1 File) was used for all subsequent experiments.

### Construction of HUWE1 catalytic mutant CSNK1A1^KO^; HUWE1^C4341R^ cell lines by base editing

An oligonucleotide encoding an sgRNA complementary to exon 83 of *HUWE1* (S2 File) was designed to include the targeted nucleotide within the editing window of the base editor ABEmax (positions 4-8 in the protospacer) using BE-Hive (https://www.crisprbehive.design), an online base editing sgRNA design tool [52], and cloned into MLM3636 according to a published protocol (Joung Lab gRNA cloning protocol: https://media.addgene.org/data/plasmids/43/43860/43860-attachment_T35tt6ebKxov.pdf). A transfection mix was prepared by diluting 750 ng pCMV-ABEmax-P2A-mCherry and 250 ng MLM3636-HUWE1-C4341R-sgRNA1 in 50 µl Opti-MEM I, adding 2 µl of X-tremeGENE HP and incubating for 20 min at RT. CSNK1A1KO cells were reverse-transfected in a well of a 24-well plate by overlaying 0.5 ml of CGM 2 (without antibiotics) containing 6 x 105 cells over the 50 µl of transfection mix. Cells were passaged to a 6 cm dish ~24 hr post-transfection, using 150 µl of Trypsin-EDTA (0.25%) (Thermo Fisher Scientific Cat. # 25200056) to detach them. Three days post-transfection, single transfected (mCherry+) cells were sorted into 96-well plates containing 200 µl of CGM 2 per well and grown undisturbed for 16–18 days. Cells were expanded and genotyped as described above.

### Targeting *HUWE1* by CRISPR/Cas9 in HEK293T-7TG and HEK293T-7TG CSNK1A1^KO^ cells

HEK293T-7TG is a clonal cell line derived from HEK293T cells that contains a fluorescent WNT reporter. HEK293T-7TG CSNK1A1^KO^ is a clonal cell line derived from HEK293T-7TG cells in which CSNK1A1 has been knocked out. Construction of both cell lines will be described elsewhere.

Oligonucleotides HUWE1-IVT-2503-F and HUWE1-IVT-2503-R encoding sgRNAs complementary to exon 6 of *HUWE1* (S2 File) were designed using sgRNA Scorer 2.0 [53] and cloned into LentiCRISPRv2-mCherry previously digested with BsmBI. Clonal HEK293T-7TG and HEK293T-7TG CSNK1A1^KO^ cell lines in which HUWE1 was targeted by CRISPR/Cas9 were established by transient transfection with LentiCRISPRv2-mCherry containing the sgRNAs followed by single cell sorting as follows. ~ 24 hr before transfection, 8 x 10^4^ HEK293T-7TG or HEK293T-7TG CSNK1A1^KO^ cells per well were seeded in 24-well plates and grown in CGM 1. On the day of transfection, CGM 1 was replaced with 450 µl of antibiotic-free CGM 1. 50 µl of a transfection mixture containing 500 ng LentiCRISPRv2-mCherry and 1 µl of X-tremeGENE HP DNA Transfection Reagent (Millipore Sigma, Cat # 06366236001) prepared in OptiMEM were added dropwise. ~ 24 hr post-transfection, cells were passaged to a 6 cm dish, and ~72 hr post-transfection, single transfected (mCherry^+^) cells were sorted into 96-well plates containing 200 µl of CGM 1 media per well and grown undisturbed for 16 days. Single colonies were expanded by passaging to 24-well plates, and 10 μl of cell suspension were reserved for genotyping.

For genotyping, genomic DNA was extracted by adding 4 volumes of QuickExtract DNA Extraction Solution (Epicentre, Cat # QE09050) to the cells. Extracts were incubated for 10 min at 65°C, 3 min at 98°C, and 5 µl were used as input for PCR amplification of the *HUWE1* target site in 15 µl reactions containing 1X LongAmp Taq reaction buffer, 300 mM of each dNTP, 400 nM of each of the flanking primers PS1057-NGS-F and PS1057-NGS-R (S2 File) and 0.1 units/µl of LongAmp Taq DNA polymerase (NEB Cat. # M0323L). In a second amplification step, complete Illumina adapter sequences (F: 5’-AAT GAT ACG GCG ACC ACC GAG ATC TAC AC < 8 bp barcode> AC ACT CTT TCC CTA CAC GAC GCT CTT CCG ATC* T-3’; R: 5’-CAA GCA GAA GAC GGC ATA CGA GAT < 8 bp barcode> G TGA CTG GAG TTC AGA CGT GTG CTC TTC CGA TC*T-3’; * indicates a phosphorothioate (PTO) linked base) were added and the amplicons were sequenced on the MiSeq system (Illumina). FASTQ sequencing files were analyzed using the branch 1.1 version [54] of a previously described analysis pipeline (https://github.com/rajchari2/ngs_amplicon_analysis). Total (dark blue) and out-of-frame (light blue) mutation rates were calculated and plotted (S6 Fig).

### Preparation of lentivirus, lentiviral transduction, and selection of *HUWE1* KD and AXIN1-overexpressing polyclonal cell populations

The transfer plasmid used to generate *HUWE1* KD cell lines by CRISPRi was Lenti-(BB)-EF1a-KRAB-dCas9-P2A-BlastR. The transfer plasmid used to generate cell lines overexpressing AXIN1 was pLenti6.2-V5-EXP-N-TERM-S-FLAG-N-hAXIN1. ~ 24 hr before transfection, 21 x 10^6^ HEK293T cells were seeded in 20 ml of CGM 1 without antibiotics in a T-175 flask. A transfection mixture was prepared by diluting 9.3 µg of transfer plasmid, 7 µg of pMDLg/pRRE, 7 µg of pRSV-Rev, 4.66 µg of pMD2.G, 1.05 µg pCS2-YFP (as a cotransfection marker), and 87.15 µl of 1 mg/ml polyethylenimine (PEI) in a final volume of 1 ml serum-free DMEM. The mixture was incubated for 20 min at RT and added to the culture media in the flasks. The day after transfection, the media was replaced with 18 ml of CGM1 containing a total of 20% FBS without antibiotics. ~ 48 hr after transfection, the media was collected (first viral harvest), centrifuged at 1000 x g for 5 min to remove cell debris, and the supernatant was reserved at 4°C. 18 ml of fresh media were added to the flask of cells. ~ 72 hr after transfection, the media was collected (second viral harvest), centrifuged as before, and the supernatant was pooled with the first viral harvest. The pooled supernatant was filtered through 0.45 µm filters (Acrodisc syringe filters with 0.45 µm Supor membrane, Pall Corporation Cat. # 4654). The filtered media containing lentiviral particles was aliquoted, snap frozen in liquid nitrogen, and stored at -80°C.

For smaller scale preparations of the lentivirus used for AXIN1 overexpression, the above protocol was followed but the lentivirus was prepared using 293FT cells in T-25 flasks and all quantities and volumes were scaled down by ~1/7. 3 x 10^6^ 293FT cells were seeded in 5 ml of CGM 1 without antibiotics in a T-25 flask. A transfection mixture was prepared by diluting 1.33 µg of transfer plasmid, 1 µg of pMDLg/pRRE, 1 µg of pRSV-Rev, 0.66 µg of pMD2.G, 0.15 µg pCS2-YFP (as a cotransfection marker), and 69.4 µg/ml PEI in a final volume of 180 µL serum-free DMEM. The mixture was incubated for 20 min at RT and added to the culture media in the flasks. The day after transfection, the media was replaced with 2.5 ml of CGM 1 containing 20% FBS without antibiotics and the viral supernatants were collected and processed as described above.

Approximately 24 hr before transduction, 2.5 x 10^5^ HAP1-7TGP cells or derivatives thereof were seeded in a 6-well plate. Cells were transduced by adding 1 ml of lentivirus-containing supernatant mixed with 1 ml of CGM 2 and 4.4 µg/ml polybrene. ~ 24 hr post-transduction, cells were passaged to 10 cm dishes and selected with 8 µg/ml blasticidin in CGM 2 for ~96 hr. Untransduced cells from each genetic background were treated in parallel with 8 µg/mL blasticidin to ensure that all cells were killed by the time selection of transduced cells was complete.

### Analysis of WNT reporter fluorescence

To measure WNT reporter activity in HAP1-7TGP cells or derivatives thereof, ~ 24 hr before treatment cells were seeded in 24-well plates at a density of 8 x 10^4^ per well and grown in 0.5 ml of CGM 2. Cells were treated for 24 hr with the indicated concentrations of WNT3A CM diluted in CGM 2. Cells were washed with 0.5 ml PBS, harvested in 150 µl of Trypsin-EDTA (0.05%) (Thermo Fisher Scientific Cat. # 25300054), resuspended in 450 µl of CGM 2, and EGFP fluorescence was measured by FACS on either a SA3800 Spectral Cell Analyzer (Sony Biotechnology) or a CytoFLEX S Flow Cytometer (Beckman Coulter). Typically, fluorescence data for 5,000–50,000 singlet-gated cells was collected and, unless indicated otherwise, the median EGFP fluorescence ± standard error of the median (SEM = 1.253 s/n, where s = standard deviation and n = sample size) was used to represent the data.

To measure WNT reporter activity in cells treated with the GSK3A/B inhibitor CHIR-99021, ~ 24 hr before treatment cells were seeded in 6-well plates at a density of 0.5 x 10^6^ per dish. Cells were treated the following day with 10 µM CHIR-99021 (CT99021) (Selleckchem Cat. # S2924) or an equivalent volume of DMSO vehicle diluted in CGM 2 for 48 hr, replacing the media with fresh CHIR-99021 or DMSO in CGM 2 after 24 hr of treatment. Cells were washed with 2 ml PBS, harvested in 0.5 ml of 0.05% Trypsin-EDTA, resuspended in 1.5 ml of CGM 2, and EGFP fluorescence was measured as described above.

### Cell viability assay

Cells were seeded in triplicate in 96-well plates at a density of 15,000 viable cells/well and grown in 100 μL of CGM 2. After 12 or 24 hr, the cells were harvested by adding 100 μL of CellTiter-Glo 2.0 reagent per well (Promega Cat. # G9241) and were incubated at room temperature in the dark for 10 min with gentle shaking. The suspension was transferred to an opaque, white 96-well plate. Luminescence was measured in a SpectraMax iD3 (Molecular Devices) multimode plate reader to determine the amount of ATP released from metabolically active (viable) cells.

### Analysis of cell growth

Cells were seeded in 6-well plates at 3 × 10^5^ cells/well in CGM 2 and placed in the Incucyte live-cell imaging and analysis system (Satorius) housed in a humidified incubator set to 37°C and 5% CO_2_. Images were captured in phase mode at five timepoints: 12, 24, 48, 72 and 96 hr after seeding. The number of cells at each timepoint was determined using the basic analyzer function of the Incucyte 2022A software. For each cell line, the number of cells at each timepoint was normalized to the number of cells at 24 hr to compensate for any differences in seeding and/or viability.

### Quantitative (q)RT-PCR analysis

Approximately 24 hr before treatment, cells were seeded in 24-well plates at a density of 3 x 10^5^ per well and grown in 0.5 ml of CGM 2. Cells were treated for 24 hr with 50% WNT3A CM in CGM 2 where indicated. The medium was removed, cells were washed once with PBS and harvested in 400 µl of TRIzol Reagent (Thermo Fisher Scientific Cat. # 15596018). Extracts were processed according to the manufacturer’s protocol, taking the appropriate precautions to avoid contamination with nucleases, and total RNA was resuspended in 20 µl of DEPC-treated water (Thermo Fisher Scientific Cat. # AM9920). To synthesize cDNA, 125 ng of RNA were diluted in 2 µl DEPC-treated water and incubated with 0.25 µl 10X ezDNase buffer and 0.25 µl ezDNase enzyme for 5 min at 37°C to digest DNA contaminants. After DNase treatment, 1.5 µl of DEPC-treated water and 1 µl of SuperScript IV VILO Master Mix (Invitrogen Cat. # 11766500) were added and the reaction was incubated for 10 min at 25°C, 10 min at 50°C, and 5 min at 85°C. For each primer pair, a cDNA dilution series from a representative sample was analyzed to ensure that target amplification was linear across a sufficiently broad range of cDNA concentrations. cDNA was diluted 1:100 in water, and 5 µl were mixed with 5 µl of Power SYBR Green PCR Master Mix (Applied Biosystems Cat. # 4367659) containing 400 nM each of forward and reverse primer (S2 File). Triplicate reactions for each cDNA and primer pair were prepared in a MicroAmp Optical 384-well Reaction Plate (Thermo Fisher Scientific Cat. # 4309849), sealed with MicroAmp Optical Adhesive Film (Thermo Fisher Scientific Cat. # 4311971) and run using standard parameters in a QuantStudio 5 Real-Time PCR System (Applied Biosystems). Thermo Fisher cloud design and analysis software (DA2) was used to calculate the average relative abundance of *AXIN2*, *RNF43*, *TNFRSF19*, or *NKD1* mRNA normalized to *HPRT1* mRNA, and fold-changes in mRNA abundance were calculated as the quotient between the experimental and reference samples, with appropriate error propagation of the respective standard deviations (SD).

### Immunoblot analysis and quantification of soluble CTNNB1 in membrane-free supernatants (MFS)

Approximately 24 hr before treatment, cells were seeded in 6 cm dishes at a density of 2.5 x 10^6^ per dish and grown in 5 ml of CGM 2. Where applicable, cells were treated for 24 hr with 50% WNT3A CM in CGM 2, or for 48 hr with 10 µM of the GSK3A/GSK3B inhibitor CHIR-99021 or an equivalent volume of DMSO vehicle (the media was replaced with fresh CHIR-99021 or DMSO in CGM 2 after 24 hr of treatment). Cells were harvested, lysed by hypotonic shock, and extracts were prepared as follows, with all handling done at 4°C. Cells were washed twice with ~5 ml cold PBS and twice with ~5 ml cold 10 mM HEPES pH 7.4. Residual buffer was removed, and 100 µl of ice-cold SEAT buffer (10 mM triethanolamine/acetic acid pH 7.6, 250 mM sucrose, 1X SIGMAFAST Protease Inhibitor Cocktail Tablets EDTA-free (Sigma-Aldrich Cat. # S8830), 25 µM MG132 (Sigma-Aldrich Cat. # C2211), 1X PhosSTOP (Roche Cat. # 04906837001), 1 mM NaF, 1 mM Na_3_VO_4_, 1 mM dithiothreitol (DTT), 62.5 U/ml Benzonase Nuclease (EMD Millipore Cat. # 70664), 1 mM MgCl_2_) were added to the cells. Cells were scraped using a cell lifter (Corning Cat. # 3008), transferred to 2-ml centrifuge tubes and disrupted mechanically by triturating 10 times. Crude extracts were centrifuged for 20 min at 20,000 x g to pellet membranes and other insoluble cellular material, and the MFS was carefully removed, avoiding contamination from the pellet. The MFS was flash-frozen in liquid nitrogen and stored at -80°C until further processing.

Extracts were thawed quickly at RT and transferred to ice. The protein concentration in the MFS was quantified with the Pierce BCA Protein Assay Kit (Thermo Fisher Scientific Cat. # 23225), using BSA as a standard, and samples were normalized by dilution with SEAT buffer. The MFS was diluted with 4X LDS sample buffer (Thermo Fisher Scientific Cat. # NP0007) supplemented with 50 mM tris(2-carboxyethyl)phosphine (TCEP), incubated for 45 min at RT or heated at 95°C for 10 min, and 30 µg of total protein were electrophoresed alongside Precision Plus Protein All Blue Prestained Protein Standards (Bio-Rad Cat. # 1610373) in 4–15% TGX Stain-Free protein gels (BioRad, various Cat. numbers) at 75 V for 15 min followed by 100 V for 1 hr and 15 min using 1X Tris/Glycine/SDS running buffer (BioRad Cat. # 1610772). Following electrophoresis, the gel was briefly activated with UV light using a Chemidoc imager (BioRad) to covalently label proteins in the gel with Stain-Free fluorochromes.

Proteins were transferred to PVDF membranes in a Criterion Blotter apparatus (Bio-Rad Cat. # 1704071) at 60 V for 2 hr using 1X Tris/Glycine transfer buffer (BioRad Cat. # 1610771) containing 20% methanol. Following transfer, the membrane was imaged using the ChemiDoc imager and the total protein in each lane was quantified by measuring the Stain-Free signal. Membranes were cut, blocked with Odyssey Blocking Buffer (Li-Cor Cat. # 927–40000), incubated with mouse anti-β-catenin or mouse anti-GAPDH primary antibodies, washed with TBST, incubated with IRDye 800CW donkey anti-mouse IgG secondary antibody, washed with TBST followed by TBS, and imaged using a Li-Cor Odyssey imaging system. Acquisition parameters in the manufacturer’s Li-Cor Odyssey Image Studio Lite software were set so as to avoid saturated pixels in the bands of interest, and bands were quantified using background subtraction. The integrated intensity for CTNNB1 was normalized to the total protein (or in some cases to the average of total protein and the integrated intensity for GAPDH) in the corresponding lane. The average ± SD normalized CTNNB1 intensity from duplicate blots was used to represent the data.

For CHIR-99021 or DMSO vehicle treated cells, ~ 24 hr before treatment cells were seeded in 6 cm dishes at a density of 2 x 10^6^ per dish and treated the following day with 10 µM CHIR-99021 or DMSO for 48 hr. The media was replaced with fresh CHIR-99021 or DMSO in CGM 2 after 24 hr of treatment.

### Immunoblot analyses of soluble HUWE1, APC and CSNK1A1 in MFS

Some of the same membranes used to blot for soluble CTNNB1 were cut and used to blot for other proteins as indicated in the same figures. Blots were incubated with rabbit anti-Lasu1/Ureb1 (HUWE1), mouse anti-APC and mouse anti-CSNK1A1 primary antibodies. The following secondary antibodies were used: for HUWE1, IRDye 680RD donkey anti-rabbit IgG, for APC, IRDye 800CW donkey anti-mouse IgG (both were imaged using a Li-Cor Odyssey imaging system) and for CSNK1A1, peroxidase AffiniPure goat anti-mouse IgG (developed using SuperSignal West Femto Maximum Sensitivity Substrate (Thermo Fisher Scientific Cat. # 34095)).

### Immunoblot analysis of total HUWE1, APC, CTNNB1, AXIN1, AXIN2, GSK3A/B and CSNK1A1 in whole cell extracts (WCE)

Approximately 72 hr before harvest, cells were seeded in 10 cm dishes at a density of 3 x 10^6^ per dish and grown in 10 ml of CGM 2. Cells were harvested in 2 ml Trypsin-EDTA (0.05%) and resuspended in 6 ml CGM 2. 10 x 10^6^ cells were centrifuged at 400 x g for 5 min, washed in 5 ml PBS, and centrifuged at 400 x g for 5 min. The supernatant was aspirated, and the cell pellets were flash-frozen in liquid nitrogen and stored at -80°C. Pellets were thawed quickly at RT and transferred to ice. All subsequent steps were done on ice. The cell pellets were resuspended in 150 µl of ice-cold RIPA lysis buffer (50 mM Tris-HCl pH 8.0, 150 mM NaCl, 2% NP-40, 0.25% deoxycholate, 0.1% SDS, 1X SIGMAFAST protease inhibitors, 1 mM MgCl_2_, 62.5 U/ml Benzonase Nuclease, 1 mM DTT, 10% glycerol), sonicated in a Bioruptor Pico sonication device (Diagenode) 4 x 30 s in the ultra-high setting, centrifuged 10 min at 20,000 x g and the supernatant (WCE) was recovered.

The protein concentration in the WCE was quantified using the Pierce BCA Protein Assay Kit. Samples were normalized by dilution with RIPA lysis buffer, further diluted with 4X LDS sample buffer supplemented with 50 mM TCEP, incubated for 45 min at RT, and 30 µg of total protein were electrophoresed alongside Precision Plus Protein All Blue Prestained Protein Standards in 4–15% Criterion TGX Stain-Free protein gels at 75 V for 15 min followed by 100 V for 1 hr and 15 min using 1X Tris/Glycine/SDS running buffer.

Proteins were transferred at 60 V for 2 hr to PVDF membranes using 1X Tris/Glycine transfer buffer containing 20% methanol, and membranes were cut and blocked with either Odyssey Intercept Blocking Buffer or TBST, 5% skim milk. Blots were incubated with rabbit anti-Lasu1/Ureb1 (HUWE1), rabbit anti-APC, mouse anti-β-catenin, rabbit anti-AXIN1, rabbit anti-AXIN2, rabbit anti-GSK3A/B, mouse anti-CSNK1A1 and mouse anti-GAPDH (as a loading control) primary antibodies, washed with TBST, incubated with Peroxidase AffiniPure anti-rabbit or anti-mouse secondary antibodies, washed with TBST followed by TBS, and developed with SuperSignal West Femto.

### Immunoblot analysis and quantification of non-phospho CTNNB1 (S33/S37/T41) and total CTNNB1 in WCE

Approximately 24 hr before treatment, cells were seeded in 6 cm dishes at a density of 2 x 10^6^ per dish. Were applicable, cells were treated for 24 hr with 50% WNT3A CM in CGM 2, or for 48 hr with 10 µM CHIR-99021 or an equivalent volume of DMSO vehicle (the media was replaced with fresh CHIR-99021 or DMSO in CGM 2 after 24 hr of treatment). Cells were harvested in 1 ml Trypsin-EDTA (0.05%) and resuspended in 3 ml CGM 2. Cells were centrifuged at 400 x g for 5 min, washed in 5 ml PBS, and centrifuged at 400 x g for 5 min. The above protocol for immunoblot analysis of total proteins in WCE was followed, except that protein samples were heated at 95°C for 10 min prior to electrophoresis, and the total protein in each lane was quantified as follows and used for normalization. Following electrophoresis, the gel was briefly activated with UV light using a Chemidoc imager (BioRad) to covalently label proteins in the gel with Stain-Free fluorochromes. Following transfer, the membrane was imaged using the ChemiDoc, and the total protein in each lane was quantified by measuring the Stain-Free signal. The blots were incubated with rabbit non-phospho (active) β-catenin (Ser33–37-Thr41), mouse anti-β-catenin or mouse anti-GAPDH (as a loading control) primary antibodies, IRDye 680RD donkey anti-rabbit IgG or IRDye 800CW donkey anti-mouse IgG secondary antibodies, and imaged using a Li-Cor Odyssey imaging system.

### Quantitative dot blot of HUWE1 in WCE

3 µl WCE containing 8 µg protein were spotted onto nitrocellulose membrane for each sample in triplicate. The membrane was allowed to dry for 15 min prior to staining with Revert 520 Total Protein Stain (Li-Cor Cat. # 926-10010) according to the manufacturer’s protocol (https://www.licor.com/documents/1o8anlg26tnwqkj135ki6bo61fy4ztmi). The membrane was imaged on the Li-Cor Odyssey M imaging system using the 520 nm channel to obtain a total protein quantification for normalization. The membrane was then blocked with Odyssey Blocking Buffer, incubated with rabbit anti-Lasu1/Ureb1 (HUWE1), washed with TBST, incubated with IRDye 680RD donkey anti-rabbit IgG secondary antibody, washed with TBST followed by TBS, and imaged using the Li-Cor Odyssey M imaging system. Acquisition parameters in the manufacturer’s Li-Cor Odyssey Image Studio Lite software were set so as to avoid saturated pixels in the dots of interest, and dots were quantified using background subtraction. The integrated intensity for HUWE1 was normalized to that for Revert 520 Total Protein Stain in the same blot, and the average ± SD from triplicate dot blots was used to represent the data. The specificity of the HUWE1 signal was confirmed by comparing dot blots of WCE from CSNK1A1^KO^ and CSNK1A1^KO^; HUWE1^KO^ cells. All normalized HUWE1 intensity values were within the linear range of a standard curve prepared from dot blots of a serial dilution of WCE from CSNK1A1^KO^ cells.

### Structural comparison of HUWE1 HECT domains

WT and C4341R mutant HECT domains (residues 4038-4374, Unitprot Q7Z6Z7-1) were predicted using the AlphaFold 3 webserver (https://deepmind.google/technologies/alphafold/alphafold-server/). The structural predictions and crystal structure were visualized and superimposed using the ‘align’ command in PyMol. For the crystal structure (PDB: 3G1N), only residues 4038-4366 of a single monomer were displayed. Cartoon displays with the catalytic C or mutant R residues shown as sticks were created using the ‘draw’ command and exported as.PNG files.

### Raw data, statistical analyses and preparation of figures

The raw data (i.e., values behind the mans, standard deviations, etc.) used to build the graphs included in the figures are shown in S3 File. Statistical analyses were performed in Excel (Microsoft). Details of the statistical tests used are given in the figure legends and shown in S3 File. Significance is indicated as ****(p < 0.0001), *** (p < 0.001), ** (p < 0.01), * (p < 0.05) or n.s. (not significant). Graphs were prepared using Prism 6 (GraphPad). Pictures of immunoblots were adjusted for contrast and brightness only when necessary for clarity using Image Studio Lite (LiCor). Images of the structural models of HUWE1 HECT domains were rendered in PyMol. Figures were prepared using PowerPoint (Microsoft). Table 1, and S1, S2 and S3 Files were prepared using Excel.

## Supporting information

S1 Fig HUWE1 and AXIN1 reciprocally regulate WNT signaling by modulating GSK3A/GSK3B-dependent CTNNB1 phosphorylation and abundance. We note that the data for WT HAP-7TGP, CSNK1A1^KO^ and CSNK1A1^KO^; HUWE1^KO^ cells is discussed in the first section of the results, while the data for CSNK1A1^KO^; AXIN1^OE^ and CSNK1A1^KO^; HUWE1^KO^; AXIN1^OE^ cells is discussed in a later section of the results subtitled “HUWE1 enhances WNT signaling by antagonizing the destruction complex.” Cells were treated with DMSO vehicle or 10 µM of the GSK3A/GSK3B inhibitor CHIR-99021 for 48 hr where indicated. (A) Immunoblots of soluble CTNNB1 in MFS, used for quantification in Fig 1B. (B) Immunoblots of non-phospho CTNNB1 (S33/S37/T41) and total CTNNB1 in WCE, used for quantification in D, E and Fig 1E. (C) Immunoblots of soluble and non-phospho CTNNB1 (S33/S37/T41) in MFS, used for quantification in D. (D) Soluble and non-phospho CTNNB1 (S33/S37/T41) abundance (CTNNB1 intensity normalized to GAPDH, from immunoblots shown in C) in MFS, and total and non-phospho CTNNB1 (S33/S37/T41) abundance (CTNNB1 intensity normalized to total protein, average from duplicate immunoblots shown in B) in WCE of the indicated cell lines, relative to CSNK1A1^KO^ cells. (E) Total CTNNB1 abundance (CTNNB1 intensity normalized to total protein, average ± SD from duplicate immunoblots shown in B) in WCE of the indicated cell lines, relative to WT HAP17TGP cells treated with DMSO.
(TIF)

S2 Fig HUWE1 enhances WNT signaling through a mechanism independent of changes in CTNNB1 abundance. (A) Genomic nucleotide and corresponding amino acid sequences comprising the WT CTNNB1 phosphodegron and the phosphodegron of CTNNB1^ST-A^ cells, with mutations indicated in red. The kinases that phosphorylate S or T residues in the phosphodegron are indicated. (B) Immunoblots of soluble HUWE1 and CTNNB1 in MFS of the indicated cell lines. The CTNNB1 immunoblots were used for quantification in Fig 2A. (C–E) Treatment of CTNNB1^ST-A^ cells with WNT3A does not promote further accumulation of soluble CTNNB1 and does not further increase endogenous WNT target gene expression. Cells were treated with 50% WNT3A CM for 24 hr where indicated. (C) Immunoblots of total CTNNB1 in WCE used for quantification in D. (D) Total CTNNB1 abundance (CTNNB1 intensity normalized to total protein and GAPDH intensity, average  ± SD from duplicate lanes of the immunoblots shown in C) in WCE of CTNNB1^ST-A^ cells treated with WNT3A CM, relative to untreated CTNNB1^ST-A^ cells. Significance was determined by unpaired t-test with Welch’s correction. (E) mRNA abundance of WNT target genes (average ± SD *AXIN2*, *RNF43*, *TNFRSF19*, or *NKD1* mRNA normalized to *HPRT1* mRNA, each measured in triplicate reactions) in CTNNB1^ST-A^ cells treated with WNT3A CM, relative to untreated CTNNB1^ST-A^ cells. (F) WNT reporter activity (median EGFP fluorescence from 5,000 singlets) for the indicated cell lines, relative to the average for CTNNB1^ST-A^ cells. Each circle represents a unique clonal cell line (determined by genotyping, S1 File), and the average of 9–12 independent clones for each genotype is indicated by a horizontal line and quantified above each group of circles. Significance was determined by unpaired t-test with Welch’s correction.
(TIF)

S3 Fig HUWE1 enhances WNT signaling through mechanisms mediated by a subset of destruction complex components including APC, AXIN1 and GSK3A or GSK3B. (A) Immunoblot analysis of total protein in WCE of the indicated clonal cell lines used for CRISPRi-mediated *HUWE1* KD in Figs 4 and S4. The “a” and “b” superscripts next to the protein names indicate which of two membranes the corresponding strips were cut from. Dashed vertical lines indicate a rearrangement of samples within the same blot. The * in the APC blot indicates a non-specific band observed with the rabbit anti-APC antibody. The AXIN1 and AXIN2 immunoblots of CSNK1A1^KO^; AXIN1^KO^ and CSNK1A1^KO^; AXIN2^KO^ cells, respectively, showed bands of lower abundance and molecular weight than their respective counterparts in WT HAP1-7TGP cells. These bands may represent residual truncated protein products, but in both cases frameshift mutations in the single allele of the respective genes (determined by genotyping, S1 File) predicted the absence of full-length, WT proteins. (B, C) GSK3A and GSK3B are functionally redundant in WNT signaling in HAP1 cells. The same cell lines were used in B and C. (B) Immunoblot analysis of total GSK3A and GSK3B in WCE of the indicated cell lines. (C) WNT reporter activity (median EGFP fluorescence from 50,000 singlets was measured for experimental duplicates of a single clone, and the average ± SD of the two measurements was calculated) relative to untreated WT HAP1-7TGP cells. Cells were treated with 50% WNT3A CM for 24 hr where indicated. (D) HUWE1 abundance, quantified by dot blots, in the clonal cell lines used for CRISPRi-mediated *HUWE1* KD in Figs 4 and S4. Total HUWE1 abundance (HUWE1 intensity normalized to total protein, average ± SD from triplicate dot blots) in WCE of the indicated cell lines, relative to WT HAP1-7TGP cells. Significance was determined by unpaired t-test with Welch’s correction. In all cases, the difference in HUWE1 abundance between each mutant cell line and WT HAP1-7TGP cells was not significant (not depicted).
(TIF)

S4 Fig HUWE1 enhances WNT signaling through mechanisms mediated by a subset of destruction complex components including APC, AXIN1 and GSK3A or GSK3B. (A and B) Polyclonal cell populations targeted for CRISPRi-mediated *HUWE1* KD with either of two HUWE1 sgRNAs (HU1 or HU2), or with SCR sgRNA control were derived for each genotype as described in Materials and methods. (A) Immunoblots of total HUWE1 in WCE used for quantification in B. The “a” and “b” superscripts next to the protein names indicate which of two duplicate membranes the corresponding strips were cut from. The dashed vertical line indicates a rearrangement of samples within the same blot. (B) HUWE1 abundance (average HUWE1 intensity normalized to either Na^+^/K^+^ ATPase or GAPDH intensity from duplicate immunoblots shown in A) in WCE of cell populations targeted with HUWE1 sgRNAs, reported as percentage of HUWE1 abundance in WCE of cell populations targeted with SCR sgRNA control. (C–F) Relative WNT target gene expression (average quantification of *AXIN2*, *RNF43*, *NKD1* or *TNFRSF19* mRNA normalized to *HPRT1* mRNA, each measured in triplicate reactions) in WT HAP1-7TGP and HUWE1^KO^ clonal cell lines, following treatment with 50% WNT3A CM for 24 hr where indicated. Each circle represents a unique clonal cell line (determined by genotyping, S1 File). For each genotype and treatment, the average value from three independent clones relative to three untreated WT clones, is indicated by a horizontal line and quantified above each group of circles. Significance was determined by paired t-test.
(TIF)

S5 Fig Regulation of WNT signaling by HUWE1 requires its ubiquitin ligase activity. (A–C) Superimpositions of the WT HUWE1 HECT domain crystal structure (PDB: 3G1N), and structural models of the WT HUWE1 and mutant HUWE1^C4341R^ HECT domains generated by AlphaFold 3. Residues 4038–4366 (crystal structure) or 4038–4374 (AlphaFold 3 predictions) of the HUWE1 HECT domain (Uniprot Q7Z6Z7-1) are shown, with the N-terminus labeled. The purple spheres represent a Na^+^ ion in the crystal structure. Red arrows indicate the catalytic C residue in the WT HECT domain, or the R residue in the mutant HUWE1^C4341R^ HECT domain. (D) Immunoblots of soluble CTNNB1 in MFS, used for quantification in Fig 5F. (E) Immunoblots of non-phospho CTNNB1 (S33/S37/T41) and total CTNNB1 in WCE, used for quantification in Fig 5G and 5H, respectively.
(TIF)

S6 Fig Quantification of CRISPR/Cas9-mediated *HUWE1* mutations in HEK293T-7TG and HEK293T-7TG CSNK1A1^KO^ cells. (A and B) Sequencing reads of the *HUWE1* locus targeted by CRISPR/Cas9 in individual clonal cell lines derived from HEK293T-7TG (A) or HEK293T-7TG CSNK1A1^KO^ (B) cells were quantified for mutations. The X-axis shows individual clones, and the Y-axis indicates the percentage of reads containing mutations. Bars in dark blue indicate the percentage of reads containing any kind of mutation (total mutations) at the targeted locus in each clone, and bars in light blue indicate the percentage of reads containing out-of-frame mutations at the same locus. In all 113 clones in which ~100% of the reads contained mutations (indicating all *HUWE1* alleles had been successfully targeted), some of those mutations were always in frame, strongly suggesting that at least one WT *HUWE1* allele is required for cell viability in HEK293T cells.
(TIF)

S1 Movie Cell growth analysis of CSNK1A1^KO^ cells. Time-lapse movie of CSNK1A1^KO^ cells from frames taken at 0, 12, 24, 48, 72 and 96 hr after seeding. The last 4 frames were used for quantification in Fig 1D.
(MP4)

S2 Movie Cell growth analysis of CSNK1A1^KO^; HUWE1^KO^ cells. Time-lapse movie of CSNK1A1^KO^; HUWE1^KO^ cells from frames taken at 0, 12, 24, 48, 72 and 96 hr after seeding. The last 4 frames were used for quantification in Fig 1D.
(MP4)

S1 File CRISPR/Cas9-engineered clonal cell lines used in this study. Single-mutant clones in which a single gene was targeted using CRISPR/Cas9 and double- or triple-mutant clones in which multiple genes were targeted using CRISPR/Cas9 are described in two separate spreadsheets labeled accordingly. When more than one clone was generated using the same CRISPR guide, the ‘Clone Name’ column indicates the generic name used throughout the manuscript to describe the genotype, and the ‘Clone #’ column identifies an individual clone. The ‘HDR Donor’ column indicates the name of the ssODN donor template used to generate some of the clonal cell lines (see Materials and methods). The ‘CRISPR guide’ column indicates the name of the guide used, which is the same as that of the oligos encoding sgRNAs (see Materials and methods, and S2 File). The ‘Genomic Sequence’ column shows 80 bases of genomic sequence (5’ relative to the gene is to the left) surrounding the target site. For each group of clones made using the same CRISPR guide (separated by gray spacers), the ‘Genomic Sequence’ column is headlined by the reference WT genomic sequence (obtained from RefSeq), with the guide sequence colored blue. The site of the double strand cut made by Cas9 is between the two underlined bases. Sequencing results for individual clones are indicated below the reference sequence. Some clones that remained WT at the targeted locus are indicated as such and were used as controls. For mutant clones, mutated bases are colored red (dashes represent deleted bases, three dots are used to indicate that a deletion continues beyond the 80 bases of sequence shown, and large insertions are indicated in brackets), and the nature of the mutation and the resulting genotype are described in the columns labeled accordingly. The figures in which each clone was used are also indicated. For double- and triple-mutant clones, the CRISPR guide used, the genomic sequence, the mutation and the genotype pertaining to each of the two or three targeted loci are designated ‘1’, ‘2’ and ‘3’ in the column headings, and are shown under green, orange and purple spacers, respectively.
(XLSX)

S2 File Oligonucleotides and primers used in this study. Oligonucleotides and primers used for generation and characterization of clonal cell lines engineered using CRISPR/Cas9 nuclease (CRISPRn), base editing, and CRISPRi, as well as those used for qRT-PCR, are described in separate spreadsheets labeled accordingly. CRISPRn, base editing and CRISPRi: the names and sequences of pairs of CRISPR guide oligonucleotides encoding sgRNAs, which were cloned into the respective vectors for each application as described in Materials and methods, are shown in columns A and B, respectively. Additionally, for CRISPRn and base editing the names and sequences of pairs of forward and reverse primers used to amplify corresponding genomic regions flanking sgRNA target sites are shown in columns C and D, respectively. Where applicable, the names and sequences of individual primers used to sequence the amplified target sites are shown in columns E and F, respectively. qRT-PCR: the names and sequences of pairs of forward and reverse primers used for qRT-PCR are shown in columns A and B, respectively.
(XLSX)

S3 File Raw data used to build graphs. The raw data used to build the graphs for the figures are shown in separate spreadsheets labeled according to their respective figure panels. The final values and statistical significance symbols used in the graphs are highlighted in yellow. In some cases, multiple different clonal cell lines were used to calculate averages for a given genotype and treatment. In those cases, the individual ‘in-house’ cell line names were used for our ease of tracking, but they can be easily correlated to the “Clone Name” and “Clone #” shown in S1 File and used throughout the manuscript.
(XLSX)

S1 Text Discussion of CTNNB1 phosphodegron phosphorylation analysis. (DOCX)

S2 Text Discussion of clonal analysis of *HUWE1* KO in clonal cell lines versus CRISPRi-mediated *HUWE1* KD in polyclonal cell populations. (DOCX)
