# Supplementary figures and images for: The ubiquitin ligase HUWE1 enhances WNT signaling by antagonizing destruction complex-mediated β-catenin degradation and through a mechanism independent of changes in β-catenin abundance
Source: PLoS Genet. 2025 May 27;21(5):e1011677. doi: 10.1371/journal.pgen.1011677 (PMC12148233; doi:10.1371/journal.pgen.1011677)

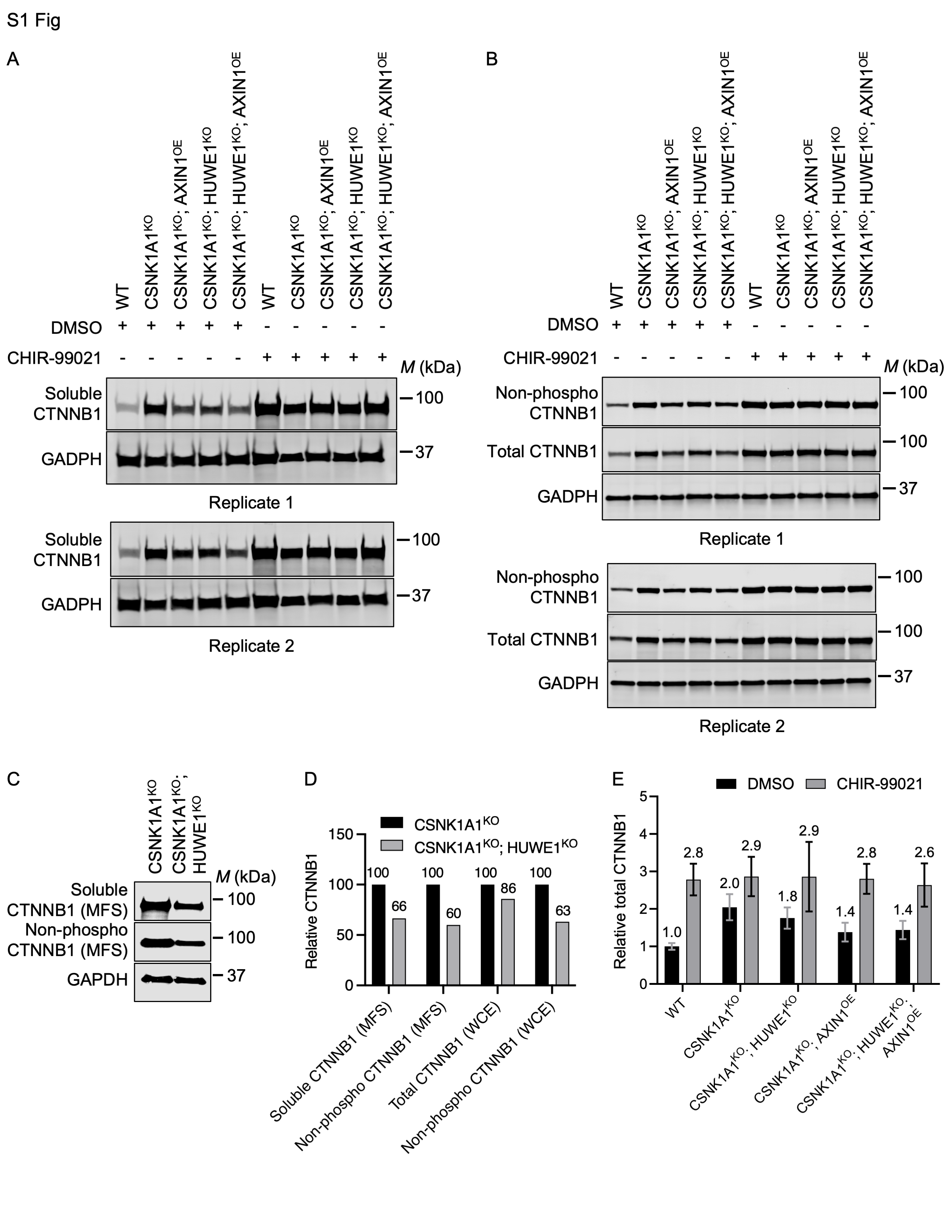

Supplement: S1 Fig — We note that the data for WT HAP-7TGP, CSNK1A1KO and CSNK1A1KO; HUWE1KO cells is discussed in the first section of the results, while the data for CSNK1A1KO; AXIN1OE and CSNK1A1KO; HUWE1KO; AXIN1OE cells is discussed in a later section of the results subtitled “HUWE1 enhances WNT signaling by antagonizing the destruction complex.” Cells were treated with DMSO vehicle or 10 µM of the GSK3A/GSK3B inhibitor CHIR-99021 for 48 hr where indicated. (A) Immunoblots of soluble CTNNB1 in MFS, used for quantification in Fig 1B. (B) Immunoblots of non-phospho CTNNB1 (S33/S37/T41) and total CTNNB1 in WCE, used for quantification in D, E and Fig 1E. (C) Immunoblots of soluble and non-phospho CTNNB1 (S33/S37/T41) in MFS, used for quantification in D. (D) Soluble and non-phospho CTNNB1 (S33/S37/T41) abundance (CTNNB1 intensity normalized to GAPDH, from immunoblots shown in C) in MFS, and total and non-phospho CTNNB1 (S33/S37/T41) abundance (CTNNB1 intensity normalized to total protein, average from duplicate immunoblots shown in B) in WCE of the indicated cell lines, relative to CSNK1A1KO cells. (E) Total CTNNB1 abundance (CTNNB1 intensity normalized to total protein, average ± SD from duplicate immunoblots shown in B) in WCE of the indicated cell lines, relative to WT HAP17TGP cells treated with DMSO. (TIF) [file pgen.1011677.s001.tif]

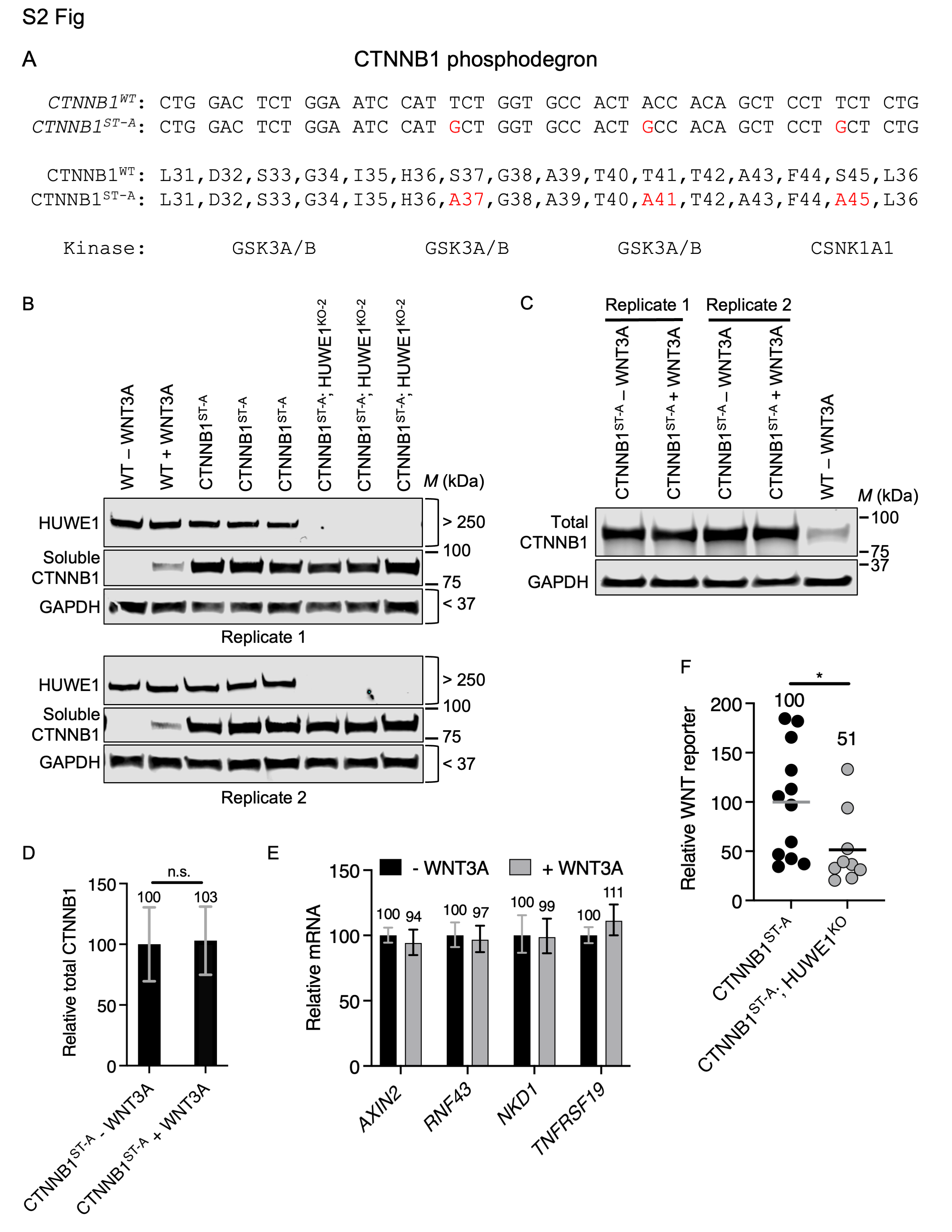

Supplement: S2 Fig — (A) Genomic nucleotide and corresponding amino acid sequences comprising the WT CTNNB1 phosphodegron and the phosphodegron of CTNNB1ST-A cells, with mutations indicated in red. The kinases that phosphorylate S or T residues in the phosphodegron are indicated. (B) Immunoblots of soluble HUWE1 and CTNNB1 in MFS of the indicated cell lines. The CTNNB1 immunoblots were used for quantification in Fig 2A. (C–E) Treatment of CTNNB1ST-A cells with WNT3A does not promote further accumulation of soluble CTNNB1 and does not further increase endogenous WNT target gene expression. Cells were treated with 50% WNT3A CM for 24 hr where indicated. (C) Immunoblots of total CTNNB1 in WCE used for quantification in D. (D) Total CTNNB1 abundance (CTNNB1 intensity normalized to total protein and GAPDH intensity, average ± SD from duplicate lanes of the immunoblots shown in C) in WCE of CTNNB1ST-A cells treated with WNT3A CM, relative to untreated CTNNB1ST-A cells. Significance was determined by unpaired t-test with Welch’s correction. (E) mRNA abundance of WNT target genes (average ± SD AXIN2, RNF43, TNFRSF19, or NKD1 mRNA normalized to HPRT1 mRNA, each measured in triplicate reactions) in CTNNB1ST-A cells treated with WNT3A CM, relative to untreated CTNNB1ST-A cells. (F) WNT reporter activity (median EGFP fluorescence from 5,000 singlets) for the indicated cell lines, relative to the average for CTNNB1ST-A cells. Each circle represents a unique clonal cell line (determined by genotyping, S1 File), and the average of 9–12 independent clones for each genotype is indicated by a horizontal line and quantified above each group of circles. Significance was determined by unpaired t-test with Welch’s correction. (TIF) [file pgen.1011677.s002.tif]

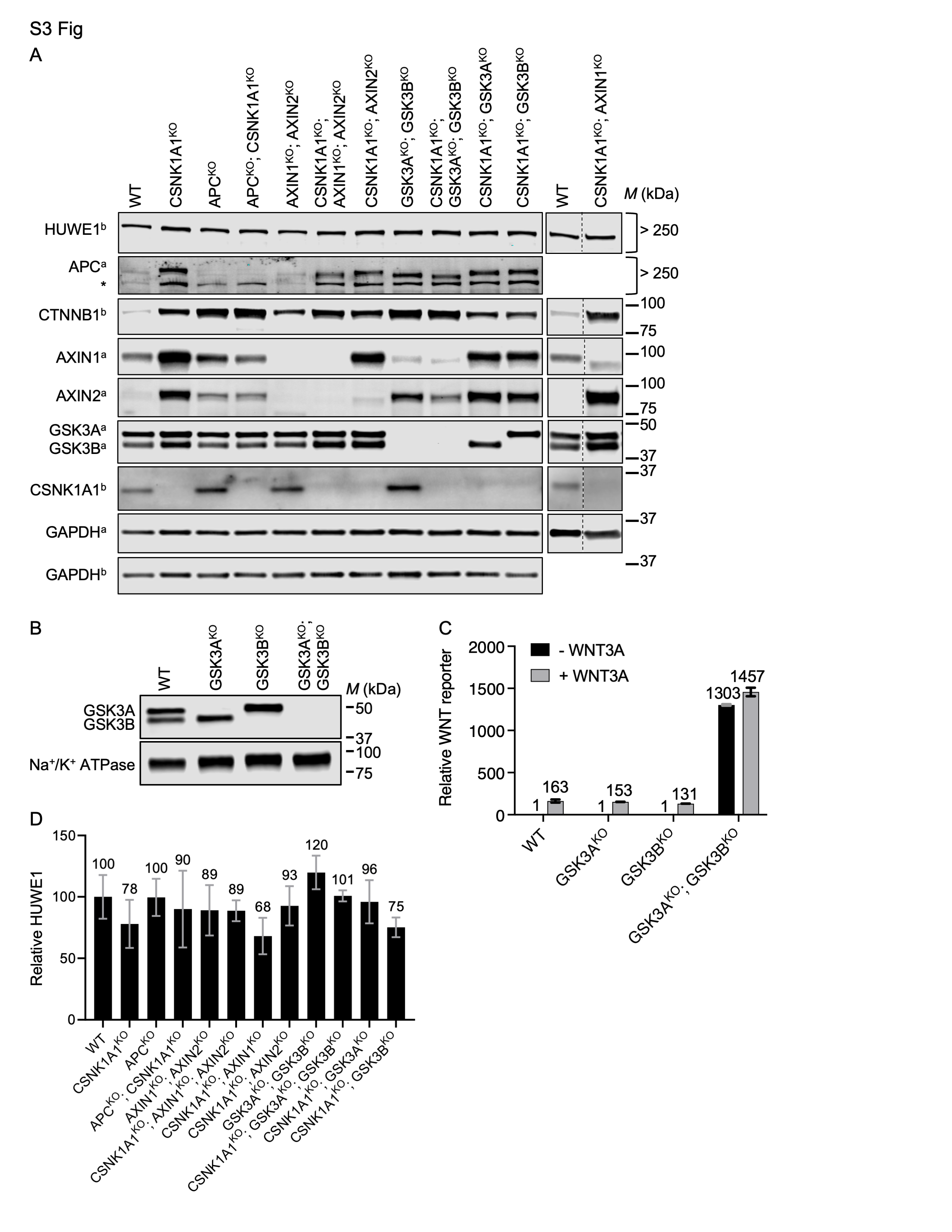

Supplement: S3 Fig — (A) Immunoblot analysis of total protein in WCE of the indicated clonal cell lines used for CRISPRi-mediated HUWE1 KD in Figs 4 and S4. The “a” and “b” superscripts next to the protein names indicate which of two membranes the corresponding strips were cut from. Dashed vertical lines indicate a rearrangement of samples within the same blot. The * in the APC blot indicates a non-specific band observed with the rabbit anti-APC antibody. The AXIN1 and AXIN2 immunoblots of CSNK1A1KO; AXIN1KO and CSNK1A1KO; AXIN2KO cells, respectively, showed bands of lower abundance and molecular weight than their respective counterparts in WT HAP1-7TGP cells. These bands may represent residual truncated protein products, but in both cases frameshift mutations in the single allele of the respective genes (determined by genotyping, S1 File) predicted the absence of full-length, WT proteins. (B, C) GSK3A and GSK3B are functionally redundant in WNT signaling in HAP1 cells. The same cell lines were used in B and C. (B) Immunoblot analysis of total GSK3A and GSK3B in WCE of the indicated cell lines. (C) WNT reporter activity (median EGFP fluorescence from 50,000 singlets was measured for experimental duplicates of a single clone, and the average ± SD of the two measurements was calculated) relative to untreated WT HAP1-7TGP cells. Cells were treated with 50% WNT3A CM for 24 hr where indicated. (D) HUWE1 abundance, quantified by dot blots, in the clonal cell lines used for CRISPRi-mediated HUWE1 KD in Figs 4 and S4. Total HUWE1 abundance (HUWE1 intensity normalized to total protein, average ± SD from triplicate dot blots) in WCE of the indicated cell lines, relative to WT HAP1-7TGP cells. Significance was determined by unpaired t-test with Welch’s correction. In all cases, the difference in HUWE1 abundance between each mutant cell line and WT HAP1-7TGP cells was not significant (not depicted). (TIF) [file pgen.1011677.s003.tif]

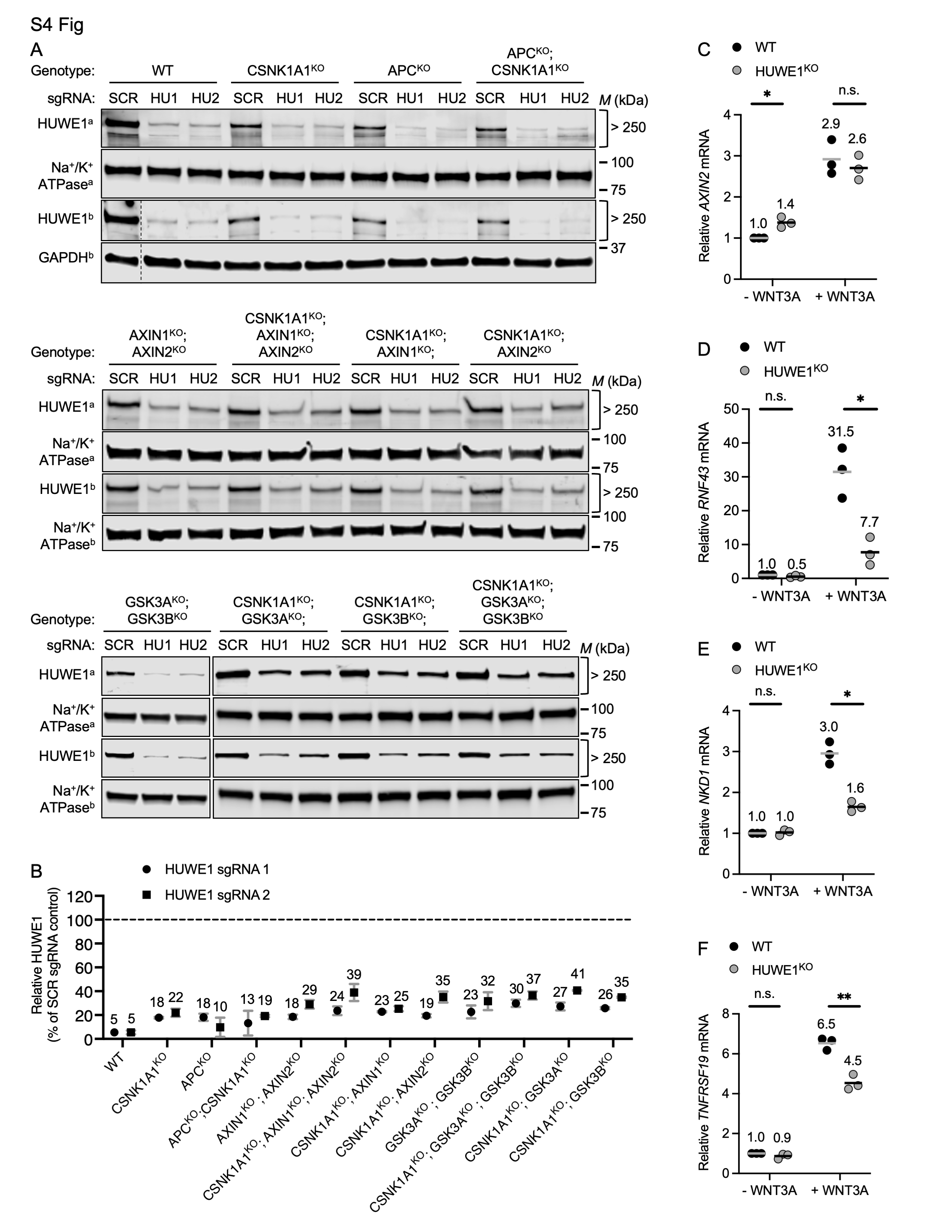

Supplement: S4 Fig — (A and B) Polyclonal cell populations targeted for CRISPRi-mediated HUWE1 KD with either of two HUWE1 sgRNAs (HU1 or HU2), or with SCR sgRNA control were derived for each genotype as described in Materials and methods. (A) Immunoblots of total HUWE1 in WCE used for quantification in B. The “a” and “b” superscripts next to the protein names indicate which of two duplicate membranes the corresponding strips were cut from. The dashed vertical line indicates a rearrangement of samples within the same blot. (B) HUWE1 abundance (average HUWE1 intensity normalized to either Na+/K+ ATPase or GAPDH intensity from duplicate immunoblots shown in A) in WCE of cell populations targeted with HUWE1 sgRNAs, reported as percentage of HUWE1 abundance in WCE of cell populations targeted with SCR sgRNA control. (C–F) Relative WNT target gene expression (average quantification of AXIN2, RNF43, NKD1 or TNFRSF19 mRNA normalized to HPRT1 mRNA, each measured in triplicate reactions) in WT HAP1-7TGP and HUWE1KO clonal cell lines, following treatment with 50% WNT3A CM for 24 hr where indicated. Each circle represents a unique clonal cell line (determined by genotyping, S1 File). For each genotype and treatment, the average value from three independent clones relative to three untreated WT clones, is indicated by a horizontal line and quantified above each group of circles. Significance was determined by paired t-test. (TIF) [file pgen.1011677.s004.tif]

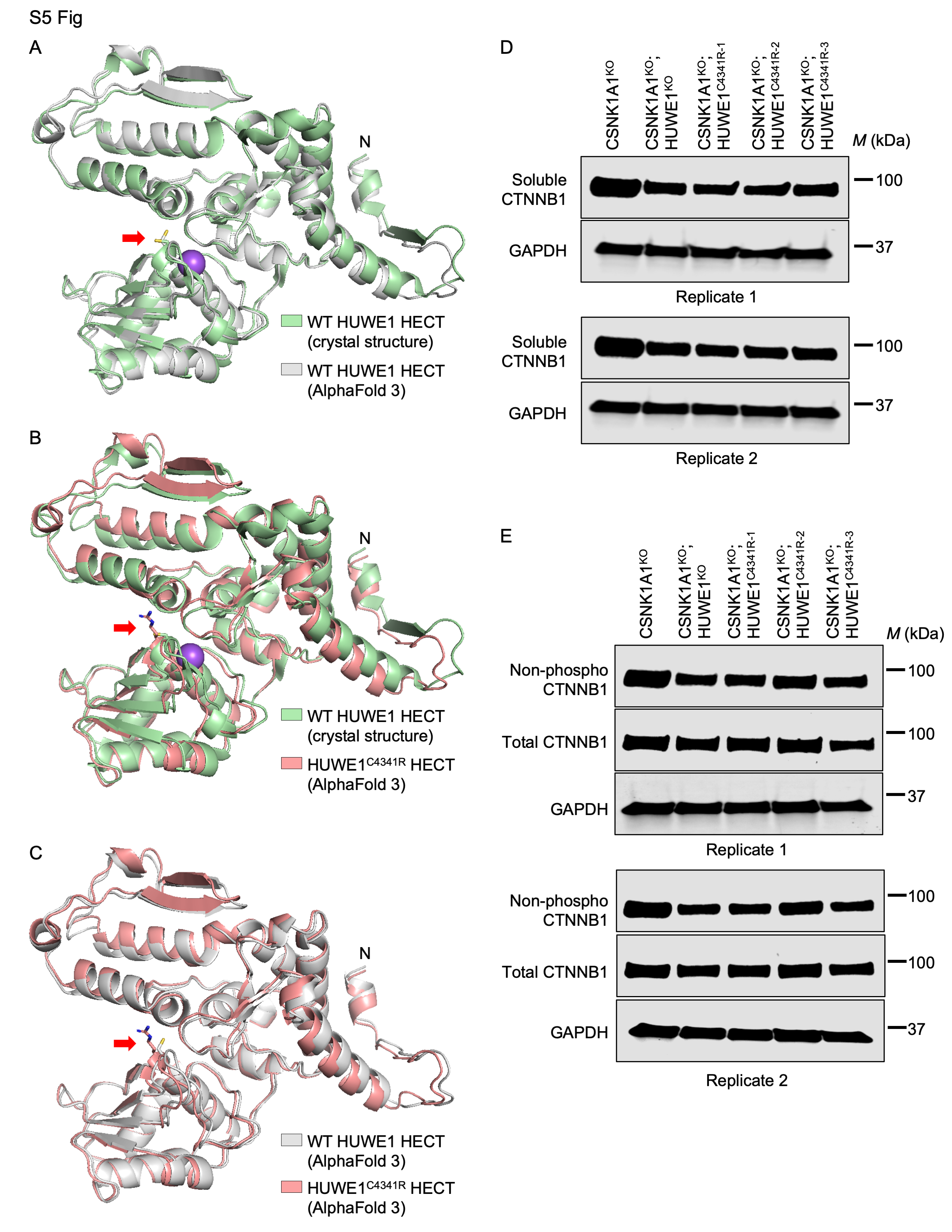

Supplement: S5 Fig — (A–C) Superimpositions of the WT HUWE1 HECT domain crystal structure (PDB: 3G1N), and structural models of the WT HUWE1 and mutant HUWE1C4341R HECT domains generated by AlphaFold 3. Residues 4038–4366 (crystal structure) or 4038–4374 (AlphaFold 3 predictions) of the HUWE1 HECT domain (Uniprot Q7Z6Z7-1) are shown, with the N-terminus labeled. The purple spheres represent a Na+ ion in the crystal structure. Red arrows indicate the catalytic C residue in the WT HECT domain, or the R residue in the mutant HUWE1C4341R HECT domain. (D) Immunoblots of soluble CTNNB1 in MFS, used for quantification in Fig 5F. (E) Immunoblots of non-phospho CTNNB1 (S33/S37/T41) and total CTNNB1 in WCE, used for quantification in Fig 5G and 5H, respectively. (TIF) [file pgen.1011677.s005.tif]

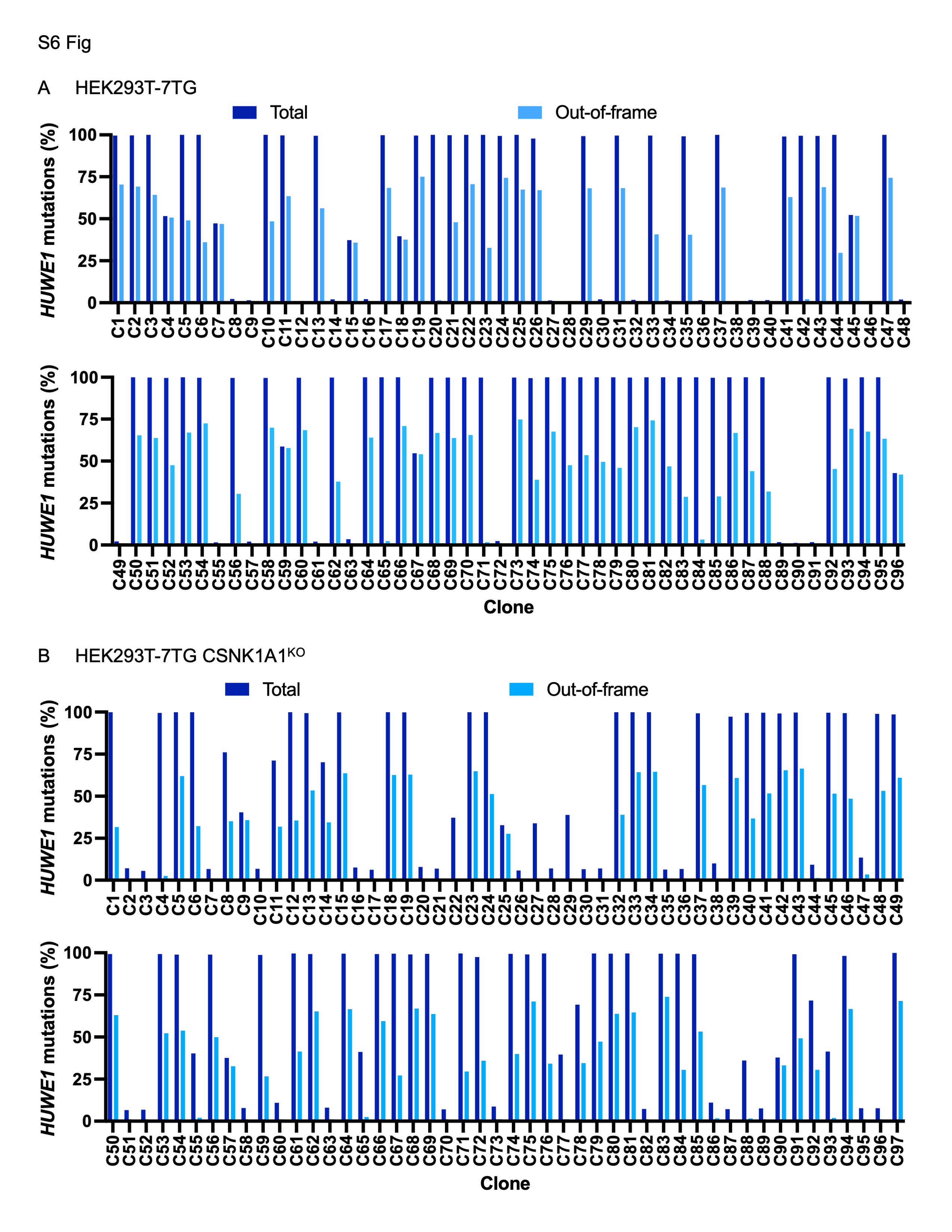

Supplement: S6 Fig — (A and B) Sequencing reads of the HUWE1 locus targeted by CRISPR/Cas9 in individual clonal cell lines derived from HEK293T-7TG (A) or HEK293T-7TG CSNK1A1KO (B) cells were quantified for mutations. The X-axis shows individual clones, and the Y-axis indicates the percentage of reads containing mutations. Bars in dark blue indicate the percentage of reads containing any kind of mutation (total mutations) at the targeted locus in each clone, and bars in light blue indicate the percentage of reads containing out-of-frame mutations at the same locus. In all 113 clones in which ~100% of the reads contained mutations (indicating all HUWE1 alleles had been successfully targeted), some of those mutations were always in frame, strongly suggesting that at least one WT HUWE1 allele is required for cell viability in HEK293T cells. (TIF) [file pgen.1011677.s006.tif]
