# Supplementary material for: The ubiquitin ligase HUWE1 enhances WNT signaling by antagonizing destruction complex-mediated β-catenin degradation and through a mechanism independent of changes in β-catenin abundance
Source: PLoS Genet. 2025 May 27;21(5):e1011677. doi: 10.1371/journal.pgen.1011677 (PMC12148233; doi:10.1371/journal.pgen.1011677)
Supplement: S1 Text — (DOCX) [file pgen.1011677.s012.docx]

**S1 Text. Discussion of CTNNB1 phosphodegron phosphorylation analysis.**

Our goal was to evaluate the extent of CTNNB1 phosphorylation by GSK3A/GSK3B at residues S33, S37 and T41 in the phosphodegron. CTNNB1 phosphorylated at S33, S37 and T41 can be measured directly by immunoblot. However, due to the rapid ubiquitylation and proteasomal degradation of the phosphorylated species, treatment with proteasome inhibitors is usually required to make accurate measurements of phosphorylated CTNNB1 [7]. Since the effects of HUWE1 on WNT signaling could conceivably also depend on proteasomal degradation of a ubiquitylated HUWE1 substrate, which would be disrupted by proteasome inhibitors, we opted for a different way in which to assess phosphorylation of the CTNNB1 phosphodegron.

CTNNB1 that is *not* phosphorylated at residues S33, S37 and T41 (we refer to this species as non-phospho CTNNB1, but it is also known as active CTNNB1 [21]) can be quantified by immunoblot. We considered quantifying non-phospho CTNNB1 in membrane-free supernatant (MFS), which is enriched for the soluble CTNNB1 fraction, or in whole cell extract (WCE), which also contains the membrane-associated CTNNB1 fraction (see Materials and methods). To evaluate how HUWE1 loss affected various CTNNB1 pools, we initially quantified soluble and non-phospho CTNNB1 in MFS as well as total and non-phospho CTNNB1 in WCE of CSNK1A1^KO^ and CSNK1A1^KO^; HUWE1^KO^ cells (S1B-S1D Figs). We found that the reduction in non-phospho CTNNB1 abundance caused by HUWE1 loss in both preparations was comparable: 40% in MFS and 37% in WCE (S1B-S1D Figs). These reductions were also comparable to the 34% reduction we measured for soluble CTNNB1 in MFS, but smaller than the 14% reduction we measured for total CTNNB1 in WCE (S1B-S1D Figs). These results suggested that most of the soluble CTNNB1 in MFS is non-phospho CTNNB1, while a fraction of CTNNB1 in WCE is phosphorylated. Therefore, non-phospho CTNNB1 in both MFS and WCE most likely represents the soluble, signaling pool of CTNNB1. We decided to measure non-phospho CTNNB1 in WCE for all further experiments because we reasoned that it would yield the most accurate overall measurement of non-phospho CTNNB1.

We also compared non-phospho CTNNB1 (Figs 1E and S1B) to total CTNNB1 (S1B and S1E Figs) in WCEs for several additional genotypes. Non-phospho CTNNB1 abundance exhibited larger changes than total CTNNB1 abundance between the various genotypes tested (Figs 1E, S1B and S1E), indicating that the immunoblot signal from the non-phospho CTNNB1 antibody only represents a fraction of the total CTNNB1 in WCEs. We surmise that the remaining fraction must be CTNNB1 partially or fully phosphorylated at S33, S37 and T41. Furthermore, inhibition of GSK3A/GSK3B with CHIR-99021 increased non-phospho CTNNB1 in WCEs to comparable levels for all the genotypes tested (Figs 1E and S1B), suggesting that the differences in non-phospho CTNNB1 abundance between genotypes in the absence of CHIR-99021 are indeed due to differences in phosphorylation of the CTNNB1 phosphodegron by GSK3A/GSK3B. We conclude that non-phospho CTNNB1 abundance in WCEs can be used to infer changes in phosphorylation of the CTNNB1 phosphodegron.
