# Supplementary material for: The ubiquitin ligase HUWE1 enhances WNT signaling by antagonizing destruction complex-mediated β-catenin degradation and through a mechanism independent of changes in β-catenin abundance
Source: PLoS Genet. 2025 May 27;21(5):e1011677. doi: 10.1371/journal.pgen.1011677 (PMC12148233; doi:10.1371/journal.pgen.1011677)
Supplement: S2 Text — (DOCX) [file pgen.1011677.s013.docx]

**S2 Text. Discussion of clonal analysis of *HUWE1* KO in clonal cell lines versus CRISPRi-mediated *HUWE1* KD in polyclonal cell populations.**

We had previously quantified the effect of HUWE1 loss on WNT signaling through an experimental scheme that we refer to as clonal analysis [15]. In this scheme, we used CRISPR/Cas9-mediated genome editing to target *HUWE1*. We isolated multiple independent clonal cell lines in which *HUWE1* had been knocked out, and multiple clonal cell lines that remained WT at the targeted locus to use as controls. We then compared several *HUWE1* KO and WT clones for WNT reporter activity and other parameters of interest (i.e. Figs 2A-2F, 3B-3F, S2F and S4C-S4F). While clonal analysis enables comparisons of true genetic null cells, it is subject to substantial inter-clonal variability, requiring the laborious isolation and analysis of many independent clones to achieve statistical significance. Isolation of multiple clones harboring *HUWE1* mutations in each of the 12 different genetic backgrounds (S3A Fig and Table 1) in which we wanted to test the effect of HUWE1 loss was unfeasible. Therefore, we used CRISPRi to knock down *HUWE1* in polyclonal cell populations, as described in the Results, and Materials and methods sections.
